# Using Human-Centered Design and Development to Create a Digital Sick Day Medication Guidance Application for People With Diabetes, Cardiovascular Disease, or Chronic Kidney Disease: Mixed Methods Study

**DOI:** 10.2196/77240

**Published:** 2025-11-27

**Authors:** Julie Babione, Sarah Gil, Chidera Okemeziem, Taylor Palechuk, Kaitlyn E Watson, Sandra Robertshaw, Nancy Verdin, Kerry McBrien, David J T Campbell, Ross Tsuyuki, Neesh Pannu, Matthew James, Maoliosa Donald

**Affiliations:** 1 Department of Medicine Cumming School of Medicine University of Calgary Calgary, AB Canada; 2 EPICORE Centre Faculty of Medicine and Dentistry University of Alberta Edmonton, AB Canada; 3 Department of Community Health Sciences Cumming School of Medicine University of Calgary Calgary, AB Canada; 4 Department of Family Medicine Cumming School of Medicine University of Calgary Calgary, AB Canada; 5 Department of Cardiac Sciences Cumming School of Medicine University of Calgary Calgary, AB Canada; 6 Department of Pharmacology Faculty of Medicine and Dentistry University of Alberta Edmonton, AB Canada; 7 Department of Medicine Faculty of Medicine and Dentistry University of Alberta Edmonton Canada

**Keywords:** sick day medication guidance, digital health, human-centred design, usability, diabetes, cardiovascular disease, chronic kidney disease

## Abstract

**Background:**

Diabetes, cardiovascular disease, and chronic kidney disease are associated with high morbidity and costs of care. Medications can reduce long-term complications but may contribute to complications such as hypoglycemia and acute kidney injury during acute illnesses. Sick day medication guidance (SDMG) could help prevent these adverse events, but evidence for effective strategies to deliver this guidance is lacking.

**Objective:**

We iteratively designed and developed a digital prototype user interface (UI) to deliver SDMG for patient self-management. The application, called “*P*reventing medication complications during *A*c*U*te illness through *S*ymptom *E*valuation and sick day guidance” (PAUSE), delivers personalized knowledge and self-management guidance directly to patients to enhance medication self-management during acute illness, with the goal of reducing preventable emergency visits and hospitalizations and improving patient outcomes during acute illness.

**Methods:**

Using a human-centered design (HCD) approach, we conducted iterative heuristic evaluation and usability testing paired with prototype revisions. Heuristic evaluation involved our team members evaluating the prototype’s UI against established criteria. We also conducted formative usability testing with 6 patients (including a patient-caregiver dyad) to provide subjective lived experience perspectives. We analyzed data deductively and pragmatically to rapidly inform subsequent iterations.

**Results:**

We identified 21 and 44 design issues through heuristics evaluation and usability testing, respectively. The development team iteratively revised the PAUSE UI prototype between evaluations, with the final design providing key user flows and integrated supports and reminders for acting on severe acute illness situations that recommend pausing certain medications.

**Conclusions:**

Using an iterative HCD approach, we designed and developed a digital health application to deliver SDMG for patient self-management. We addressed feasible technical and workflow barriers using iterative heuristic evaluations and usability testing resulting in a refined SDMG self-management prototype app for patients taking medications commonly used to treat diabetes, cardiovascular disease, and chronic kidney disease. Further research is needed to test the effectiveness of the current PAUSE app in helping people with these chronic conditions self-manage their medications during acute illness and evaluate the feasibility of integrating the app into community-based chronic disease care.

## Introduction

Diabetes, cardiovascular disease, and chronic kidney disease (CKD) are associated with high morbidity and health care costs. Effective medications are available to reduce the incidence of long-term complications; however, these same medications are associated with risks of hypoglycemia and acute kidney injury during acute illnesses, which may lead to preventable emergency room visits and hospitalizations [1-3]. These drug-related adverse events are also associated with poor outcomes including extended hospital stays and death [4]. Several international organizations recommend pausing specific medications (ie, those referred to in the SADMANS acronym: sulfonylureas, angiotensin-converting enzyme inhibitors, diuretics, metformin, angiotensin receptor blockers, nonsteroidal anti-inflammatory drugs, and sodium-glucose cotransporter 2 inhibitors) during acute illnesses [5-10]. While some pharmacists and physicians may inform patients of these recommendations, guidance delivery to patients is inconsistent, and many patients remain unaware of it [11,12]. Furthermore, despite the inclusion of sick day medication in clinical practice guidelines [13,14], evidence of effective strategies to deliver it is lacking [5,15,16].

Our previous work explored the barriers and enablers involved in providing sick day medication guidance (SDMG) to patients and found that health care providers (HCPs) often lack SDMG knowledge or confidence in providing SDMG, or do not always remember to provide SDMG [12]. On the other side, patients are often unconcerned with sick day complications or confuse sick day symptoms with chronic condition symptoms or expected side effects of their medications. SDMG guidance was also identified as something that needs to be provided when a sick day event occurs, rather than when medications are first dispensed, because patients are unlikely to remember something they were told months or years ago. Further complicating the issue, patients display a range of behaviors in medication adherence, for example, fear of medication reinitiation or stopping a medication at all [12].

If thoughtfully and rigorously designed, digital tools can offer a new and timely approach to addressing the sick day self-management needs of patients on medications qualifying for SDMG. Digital tools for SDMG should be extremely easy to use, support various levels of patient capacity, and provide the right information at the right moment and in an easily understood manner. These tools can also be integrated into existing electronic medical record (EMR) or pharmacy systems for personalized patient experiences that capitalize on the existing trustworthiness of those systems and thus addressing some of the barriers identified in the preceding paragraphs. Digital SDMG tools might also benefit from integration with wearable technologies, where smartwatch heart rate monitors, continuous glucose monitors, smart scales, and other devices could in future provide key metrics to seamlessly and passively detect a sick day for patients on certain medications and provide timely medication support [17]. To the best of our knowledge, no digital tools have been created to date that support patients with SDMG.

To further address the complex needs of patients needing SDMG as we carefully designed this digital solution, we looked to human-centered design (HCD) for guidance. HCD is a design, development, and evaluation approach that puts the user in the center of all design decisions and ensures that information is provided as clearly and concisely as possible [18-20]. In the digital space, it includes a resource-effective approach that ensures that a user interface (UI) prototype (ie, a rudimentary, interactive representation of a product’s design and functionality allowing stakeholders and users to test and validate ideas before the final product is developed) is designed with representative end user involvement and evaluated using heuristic evaluation (ie, examining a UI against established heuristic principles to identify potential problems) and usability testing (ie, observing real users interacting with the interface to identify actual usability issues) with representative end users, and revised before full product development begins [21-23].

The objective of this work was to iteratively design and refine a UI prototype to deliver SDMG for patient self-management using HCD approaches. Here, we describe the “*P*reventing medication complications during *A*c*U*te illness through** ***S*ymptom *E*valuation and sick day guidance” (PAUSE) mobile app prototype, developed as part of an overarching research program with the goal of reducing preventable emergency visits and hospitalizations, and improve outcomes during acute illness for people living with diabetes, cardiovascular disease, and CKD.

## Methods

### Human-Centered Design

**Figure 1:**
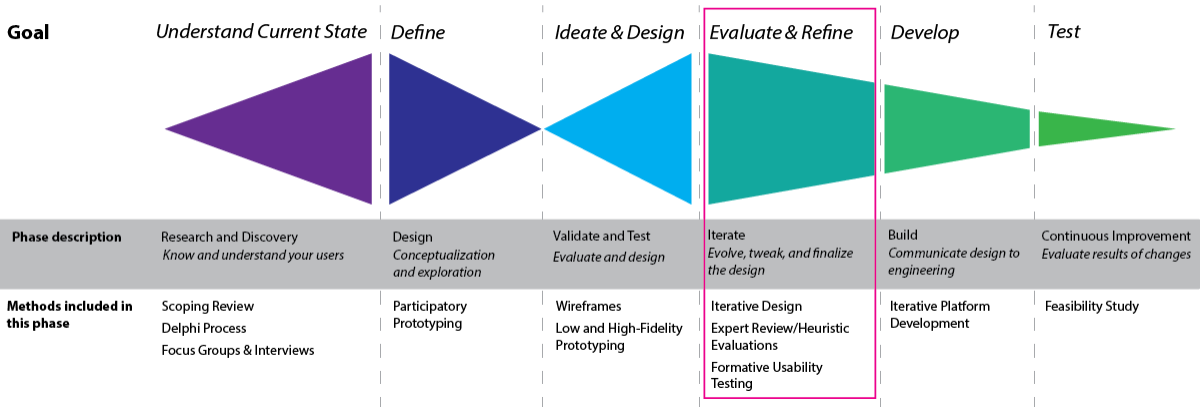
Human-centered design approach overview with methods used in this project.

A HCD approach informed our prior work addressing the goals of “Understand Current State,” “Define,” and “Ideate and Design” and have been reported elsewhere [5,11,24]. Here, we focus on the prototype’s iterative UI design and development process, specifically reporting on the heuristic evaluation and formative usability testing within “Evaluate and Refine” goal (Figure 1). These methods aimed to gather qualitative insight into the usefulness, usability, and user satisfaction of the PAUSE prototype, and provide recommendations for improving its usability, accessibility, and design.

### Heuristic Evaluation

We conducted a heuristic evaluation (also known as an expert inspection or evaluation) using an early (ie, rudimentary click-through version of the UI) prototype. The evaluation consisted of applying a set of standardized design principles (ie, heuristics), considering a severity rating of the issue, and recommending potential solutions [25,26]. These evaluations typically reveal most usability-based design problems at a relatively low-resource cost [27]. We used a standardized heuristic evaluation checklist and severity rating guide [25,26,28,29].

The heuristic checklist comprises a set of “common” usability issues inherent in many digital and physical designs [25,30] and gathered by usability engineering experts to facilitate first-pass problems before the design is put in front of representative users as part of later usability testing after making revisions in response to the heuristic evaluations. This prevents common issues from stalling users prematurely during usability testing and allows them to focus more on content and navigation-based issues that developers and designers might otherwise overlook (eg, due to the mere exposure effect—focusing on specific tasks, leading to a decrease in attention to other app elements) [31-33].

We used the severity rating scale shown in Table 1. The severity ratings help evaluators identify which issues are more or less likely to impact elements of usability (as captured in heuristic evaluations) and allow for prioritization of those elements most critical to product success [29].

We followed the pragmatic Nielsen and Norman Group’s guidance for conducting heuristic evaluations for this evaluation [28]. Five research team members (SG, JB, TP, DO, and SR) with usability and design expertise or lived-experience expertise (SR) individually and independently examined the prototype interface on various laptops, desktops, and mobile configurations to conduct this heuristic evaluation.

**Table 1:** Severity rating scale.

Rating	Meaning
1	Violates heuristic; will lead to errors
2	Violates heuristic; might lead to errors
3	Partial adherence to heuristic; will require training to learn
4	Partial adherence to heuristic; will require practice to learn
5	Complete adherence to heuristic; no problems anticipated

### Heuristic Evaluation Data Analysis

We followed the Nielsen and Norman Group’s guidance for conducting heuristic evaluations for data analysis [28]. Once independent evaluations were completed, the team met to discuss findings item by item, demonstrating issues on screen not seen by others, or where there was discrepancy in severity ratings. Two evaluators (SG and JB) then consolidated the results into a final list of the issues and group discussion notes were consolidated with group-consensus derived severity ratings. We then organized the consolidated list from highest priority to lowest priority (according to the research and design team) with proposed actionable solutions (where possible) and shared with the development team to revise the prototype UI.

### Usability Testing

We conducted formative usability testing using semistructured one-on-one interviews to identify the most likely barriers or challenges to using the PAUSE app as experienced by target end users [27,34]. We identified a sample of target end users who are living with a chronic illness and taking SDMG-qualifying medications and their caregivers through clinical staff champions who provided prospective participants with study invitation letters. Participants then completed the web-based consent forms and a demographic survey and provided contact information for further scheduling. Participants were selected to obtain maximum variation in demographic characteristics (eg, age, gender, location, and eligible conditions). The target sample size for usability testing was set at 6-8 participants in accordance with qualitative or formative usability testing guidelines, where approximately 80% of the issues can be identified in a cost and resource-effective way [34-36]. Testing sessions began with a brief presession interview aimed at collecting basic descriptive demographic information about the study participants and as a way of easing them into the interview process. This was followed by a series of predetermined representative and likely tasks that users would undertake with the prototype app (Multimedia Appendix 1), including onboarding, recording an illness and answering key questions, and responding to reminders to check in. Sessions concluded with a postsession interview with each participant, allowing them to provide subjective perspectives of their experience. Sessions were conducted in participant’s homes on their mobile phones, or when technical challenges were encountered on our study laptop where we had the mobile phone mock-up screens loaded with touch screen interaction simulations. Data were collected in the form of audio recordings and field notes.

### Usability Testing Data Analysis

Audio recordings of sessions were transcribed and added to field notes that were together analyzed using a deductive content analysis approach [37,38]. This deductive analysis approach was selected to match the practical needs of the collaborating software development team, allowing for rapid synthesis and communication of results for further iteration. We chose the predetermined categories: SDMG content, layout and presentation, interactivity, content (other), functionality and user experience, navigation and workflow, visual elements, medical terminology, and target audience. These categories informed different elements of changes needed for development to move forward. NVivo qualitative analysis software (version 14; Lumivero) was used to facilitate data management and analysis. To include our patient partners as coanalysts and accommodate the dual purpose of usability testing data (ie, academic publication and practical use in rapid turnaround development feedback), coding was completed somewhat unconventionally, although with rigor. A single coder (JB) with access to NVivo coded the transcripts according to smallest units of meaning and initially organized them into the predetermined categories as listed previously. Within these categories, issues were inductively identified (ie, if issues were repeatedly coded within a transcript or between transcripts, they were grouped together). In parallel, to help patient partners and others who did not have access to NVivo identify issues using easier to understand language, we created an Excel sheet with similar headings, which the other research team members used to organize quotes they found in the transcripts to capture issues: (1) ease of understanding (purpose of the app, context the app is used in, and perceptions of app rationale), (2) on-screen content and workflows (anything to do with the UI and content of symptoms listed), (3) performance and efficiency (functionality, How complex the app is to use? Is the app cumbersome? Is the app easy to navigate?), (4) SDMG and adoption (Will they use the advice given? Is it credible? Would they use the app for managing their health?), and (5) parking lot (anything deemed important, interesting, or relevant but unsure how to categorize).

The entire analysis team then met and discussed findings and decided on prioritization and suggested solutions, with JB live cross-checking to confirm analysis alignment between NVivo and Excel coding. The report to the development team was then written based on both analyses with the consensus priorities and recommended solutions, with this manuscript based on that full report.

### Ethical Considerations

Ethical approval for this study was obtained from the University of Calgary Conjoint Health Research Ethics Board (REB20-1989). Informed consent was obtained at all stages involving human subjects and the ability for participants to opt out were provided prior to deidentification and analysis. Any protocol modifications were submitted to the ethics review committee and enacted only after approved. Privacy and confidentiality were maintained throughout the study by all research team members (eg, interview transcripts removed any identifying information and any identifying information used for session booking was kept separated from study materials at all times). Only deidentified data were used and data were accessible only to the research team. Usability testing participants were compensated with a CAD $25 (approximately US $18.50) gift card. No conflicts of interest were declared.

## Results

### Heuristic Evaluation

A total of 21 design issues were documented and are summarized in Multimedia Appendix 2, with some highlights described in Table 2 and the accompanying text description. A full description of all the results is provided in Multimedia Appendix 3.

The analysis of the heuristic evaluation results was practical and focused on real-world application. The categories used in the analysis were decided in advance to facilitate communication with the app developers as these categories would be familiar to them. We provide a summary of the results in Table 2.

**Table 2:** Heuristic evaluation results showcasing the issues identified in the first iteration prototype UI^a^ design.^b^

Issue		Recommendation
**Navigation and workflow**		
	Workflow is incorrect in multiple areas (1): Users experience navigation issues, as some selections do not link to the correct page, selections were preselected, and buttons did not function correctly.	Remove all preselected options, ensure that all pages advance along the correct pathway, verify that the back button functions as users expect, and ensure that users can access the right pages at the right time.
	Always end up on the onboarding screen (3): After the onboarding process is completed when users open the app or press the home button, users return to the onboarding page and need to sign in again.	After completing onboarding, users should open the app directly to the home page without needing to sign in each time or view the rotating banner.
	No history or archive of advice (1): There is no way for users to revisit past advice, and the information is not saved requiring users to reenter their details. Users wish to share recommendations with their health care providers, 911/811 operators (ie, emergency dispatch), or urgent care doctors without relying solely on memory.	Create an archive section in the app where users can revisit their symptoms and the advice given.
	Onboarding process is disjointed and confusing (3): Alignment in lists is off, and there is no consistency across the screens. Specifically, screen 1 of 5 lacks organization for the home-monitoring options.	Organize options into categories, for example, “Screen 1 of 5” should have options grouped according to body areas. Ensure that the app has a consistent look and feel.
**Layout and presentation**		
	Remove resources from the check-in page (2): Users found the option to read resources when checking in distracting. The app must be clear and concise, as users will access it when unwell.	Delete resources from the check-in page and create a dedicated resources page that users can access at their convenience. Overall, to improve clarity and user experience, remove unnecessary information.
	App has dead ends and lacks user feedback (2): At the end of the onboarding process, there is no indication that it is complete. When users receive advice from the app, there is no option to exit the screen, no “X” button or “OK” button.	Avoid dead ends; users must be able to exit pages and return to the home page or previous page. User feedback is needed so that users know when they have completed a task. Suggest showing a “Thank You” message, providing a “Home” or “OK” button, or offering an option to close the page.
	Important information lost in blocks of text (3): Important information is overlooked when presented in large blocks of text.	Important information or calls to action should be separated from large blocks of text by placing them on their own line, without using brackets. Ensure that text formatting and presentation of information are consistent across all screens.
**Visual elements**		
	Confusion regarding icons in the navigation bar (3): Users are confused about the icons in the bottom navigation bar and are uncertain about their destination within the app.	Enhance the clarity of the icons by using more familiar designs. In addition, consider adding a “tooltip” resource to explain the icons to users.
	Red and green color blindness issue (3): The app uses red and green colors to highlight decisions and important areas. People who are red-green color-blind may face challenges.	Update the branding and colors to match the PC health app. Ensure that visuals are color-blind–friendly.
**Functionality and user experience**		
	Rotating banner difficult to read (3): Text on the sign in page continuously scrolls, and users cannot pause to read the information. The banner includes visual indicators (blue circles) that suggest that users can interact with it.	Remove the scrolling banner and add “Next” buttons to allow users to read the information at their own pace. Eliminate circles at the bottom of the page and replace with the “Next” button. Do not show the banner every time a user logs in; instead, place that information in the resource section.
	Communication choices during onboarding (3): When selecting notification preferences, users like the option to choose both text and email notifications. Users would prefer to finish setting up their profile before they answered the communication question.	Allow users to choose both text and email or add an option for “text and email.” Move the notification options screen to the end of the onboarding process.
	Promote user engagement (2): Language used in the app does not encourage user engagement.	Create messaging that encourages user engagement. Instead of saying, “Contact your healthcare provider if any symptoms arise,” rephrase it to: “Check in the next time you’re feeling worse than usual.” Similarly, change “Day 1 Follow-up” to: “Tap here to check-in.”
	Ensure app is tailored to user (3): During onboarding, users are asked about medications they take and whether they have home monitoring devices. When users enter their symptoms, they are asked the same questions again. Example, user is asked whether they take insulin at onboarding and then given advice that says, “if you take insulin.”	After users enter their symptoms, they should not be asked what home devices they use; instead, they should be asked questions about their device measurements. For questions regarding ketones, ensure that the inquiries reflect the type of ketones users measure, or provide an “n/a” option. Users should be directed to the insulin advice page only if they indicate that they take insulin. Also suggest removing the phrase “if you take insulin.”
	Design of the “My medications” page cluttered and confusing (3): Layout of the “My medication page” is challenging to read due to the amount of information. It is also not clear whether users edit individual medications or the entire page.	Medications should be listed clearly, and the “verified” label should be removed from public view. Each medication listed should include its own edit icon.
**Interactivity**		
	Users can turn all buttons on or off (3): When users respond to questions using “Yes” or “No” buttons, they can click on both responses, even if the question specifies that only 1 answer is allowed.	When a response requires only 1 choice, ensure that only 1 option can be selected.
**Medical terminology and SDMG^c^ details**		
	Users are confused about medical advice for symptoms (3): Users feel confused about why symptoms come with varying medical advice. They do not understand the reasons for contacting an HCP^d^, what “sick day” medications are, or why they should pause certain medications.	Suggest creating a FAQ^e^ resource that explains why users need to contact their HCP, what constitutes a sick day, and why these medications should be paused.
	Missing information to provide proper NSAIDs^f^ advice (3): During onboarding (screen 4/5), users are asked whether they take NSAIDs but not how frequently they take them.	Add a question asking users how frequently they take NSAIDs (never, rarely, occasionally, and regularly). Provide proper advice for the frequency the user selects (never, rarely, and occasionally do not receive SDMG but regularly does).
**Language**		
	“Educational Resources” as a title adds pressure (3): The phrase “Educational Resources” can be paternalistic and create a sense of top-down pressure. This suggests that the user needs to be educated or they lack knowledge about their medications or illness.	Change the heading to “Resources” to reduce top-down pressure, as this term indicates that users can access additional information as needed.
**Consistency**		
	Design of medication advice page (2): On the “Medications to Stop” page, the display of listed medications resembles selection buttons.	Ensure that the medications are listed in a way that does not suggest that they can be selected. Maintain consistency in how lists and selection options are displayed throughout the app.
	Display of the user’s name varies across screens (3): The user’s name is displayed on some screens and not on others.	Only display the user’s name on their profile page, and check-in and follow-up screens.
	App design lacks consistency (1): There is a lack of consistency throughout the app, including page designs, button appearances, navigation between pages, and the use of icons.	Review all content for any spelling and grammatical errors, and make sure that design elements, including buttons and selection options, remain consistent throughout the app.

^a^UI: user interface.

^b^The number in brackets behind each issue is the consolidated severity rating.

^c^SDMG: sick day medication guidance.

^d^HCP: health care provider.

^e^FAQ: frequently asked question.

^f^NSAIDs: nonsteroidal anti-inflammatory drugs.

The heuristic evaluation identified most issues in the categories of navigation and workflow (19%), Functionality and user experience (24%) primarily related to UI layout and interaction. Evaluators assessed the app from the perspective of a patient experiencing a relevant sick day, focusing on heuristic elements such as memory recall, consistency, and clarity of language. To support urgent care scenarios, evaluators recommended ensuring that prior advice, such as recommendations to contact emergency or urgent care assistance, could be retrieved and shared with care providers to aid the urgent care process. A notable issue was inconsistent visual design, where similar-looking buttons and visuals made it unclear what was interactive. Given the importance of seamless screen flow in delivering the correct SDMG, evaluators identified several issues for resolution before patient testing. Evaluators recommended additional adjustments to improve usability including removing distracting elements such as additional resources from the assessment workflow to prevent errors and organizing screen content more clearly to avoid confusion.

Evaluators also reviewed language, terminology, and tone to ensure a supportive and user-friendly experience. Suggested changes included (1) removing the term “Educational” from “Educational resources” as this could be perceived as paternalistic, (2) ensuring consistency between the patient’s profile-building questions (which may not be needed if this information is imported from an existing EMR or pharmacy platform), and (3) refining the prompt language to encourage users to check in with the app when they are experiencing manageable sick day symptoms.

### Usability Testing

A total of 6 participants (5 patients and a patient-caregiver dyad) who spoke and read English completed the usability testing (Table 3). The participants were older than 40 years, educated, and with a 60%/40% split between males and females. An eHEALS (eHealth Literacy Scale) mean score of 30.4 (SD 6.8) indicates a moderate to high level of perceived eHealth literacy, meaning individuals generally feel confident in finding, evaluating, and using web-based health information [39, 40]. A mean System Usability Scale score of 74.2 (SD 9.2) with median score of 73.8 suggests an above average perceived usability of the prototype [41, 42]. The results indicated a highly positive receptivity and anticipated usefulness of the PAUSE app; however, the app’s purpose and target audience needed to be refined for optimal effectiveness.

A total of 44 priority-ranked issues were identified to be addressed and resolved prior to software development (Multimedia Appendix 4). A full description of all results is shown in Multimedia Appendix 5. Most issues related to navigation and workflow and interactivity were resolved in the design iteration prior to usability testing, allowing the focus to shift to issues identified by patients and the caregiver with patient dyad with lived experience. Table 4 summarizes the usability testing results, and Multimedia Appendix 3 provides the full results with screen captures to provide further details.

Usability testing identified issues in navigation and workflow, layout and presentation, visual elements, functionality and user experience, interactivity, medical terminology, SDMG content, (general) content, (confusions around) target audience, and in a few cases branding. The research team discussed and prioritized issues and then discussed issues again with the development team to identify appropriate solutions.

We noted a few issues related to navigation and workflow as residual issues identified previously through heuristic evaluations; however, usability testing participants identified new issues as well, specifically around the app’s contextual workflow. Questions arose concerning participants’ previous experiences with clinician reluctance to discuss medication dosage changes over the phone, prompting the research team to confirm existing assumptions that this capability exists in most local community settings. One participant also noted that some at-home sick day situation management approaches require some preparation and planning, which a checklist included within the app’s resources section might facilitate. Another participant asked whether the app might inform their doctor about an ongoing sick day situation. This was deemed potentially complex and might be better for a future iteration.

Within layout and presentation, participants using smaller mobile phones were unable to see the “next” button and there were no suggestions that there was additional content further down (out of sight). A few participants also tried to use the app in landscape mode, which we noted was beyond the capability of the prototype UI; however, this will be something that should be monitored or even addressed (if possible) during app development. On the same topic of screen orientation, the narrow portrait orientation made lengthy text difficult to read. Finally, participants were challenged to identify medications based only on names, asked for pill pictures, and expressed concern about the absence of dosage information.

Several users also mentioned visual elements, with emphasis on accessibility, text size and contrast, and familiar connotations (ie, red signifies alert, danger, or stop) they found confusing in this app. Several participants also found a few inconsistent color uses within the apps (ie, for buttons) to be addressed.

For proposed functionality and user experience, the prototype UI included face and fingerprint recognition as might be included for privacy and security through a modern smartphone. However, users shared challenges and frustrations with or lack of knowledge of these elements. Others found manual medication data entry cumbersome and error prone. A potential solution to this might be to pull medication data from an existing EMR or pharmacy database.

Participants identified a few issues that we classified under interactivity. Form factor preference among participants came up, with smartphones still being somewhat less familiar among older participants than computers. The smaller size of the smartphone also made screen interactions difficult for several users (particularly those with diabetes or age-related vision problems), with checkboxes and “X” (close) buttons difficult to select. Form factor will be something to consider depending on future EMR or pharmacy platform integration plans.

Medical terminology and, most notably, language related to SDMG content came to the forefront during usability testing. For example, patients did not always easily understand content created by medical experts. In some cases, we removed elements such as medication frequency changes for over-the-counter nonsteroidal anti-inflammatory drugs because of their use complexity in real-life situations (eg, daily or occasional use that varies from situation to situation) and the impact of the question in the assessment of symptom severity. Ketones were unfamiliar to those who do not explicitly track them, which should perhaps be shown only to those who do track them. A few users found “Cope with symptoms” confusing, which researchers revised to “Are you able to manage these symptoms by yourself?” The research team collected further contextual feedback that will help position the PAUSE app for pilot testing and implementation. Some users believed that as long as they did not access their health data themselves through secure online web portals that their health data would not be online, leading them to be wary of using digital technologies to access their data. Other complexities might also arise if a patient has medications filled at different pharmacies who might provide different SDMG information.

**Table 3:** Usability testing demographics.

Characteristics				Values, n (%) or mean (SD)
**Patients (n=5)**				
	**Sex, n (%)**			
		Male	3 (60.0)	
		Female	2 (40.0)	
	**Gender, n (%)**			
		Cisgender woman	1 (20.0)	
		Cisgender man	2 (40.0)	
		Prefer not to answer	2 (40.0)	
	**Age (years), n (%)**			
		41-50	1 (20.0)	
		51-60	1 (20.0)	
		61-70	1 (20.0)	
		>70	2 (40.0)	
	**Race or ethnicity, n (%)**			
		White	4 (80.0)	
		Other	1 (20.0)	
	**Population size, n (%)**			
		<500,000	3 (60.0)	
		>500,000	2 (40.0)	
	**Education, n (%)**			
		Less than university degree	1 (20.0)	
		University degree or higher	4 (80.0)	
	**Income, n (%)**			
		<$100,000^a^	2 (40.0)	
		>$100,000	3 (60.0)	
	**Employment status, n (%)**			
		Full-time employee or student	2 (40.0)	
		Retired	3 (60.0)	
	**Marital status, n (%)**			
		Single	1 (20.0)	
		Married or common law	4 (80.0)	
	**Chronic condition, n (%)**			
		Chronic kidney disease	3 (60.0)	
		Heart failure	1 (20.0)	
		Diabetes	2 (40.0)	
		Other	1 (20.0)	
	**eHEALS^b^ score, mean (SD)**			30.4 (6.8)
	**SUS^c^ total, mean (SD)**			5.6 (1.1)
**Caregivers (n=1)**				
	**Sex, n (%)**			
		Female	1 (100.0)	
	**Gender, n (%)**			
		Cisgender woman	1 (100.0)	
	**Age (years), n (%)**			
		>70	1 (100.0)	
	**Race or ethnicity, n (%)**			
		White	1 (100.0)	
	**Population size, n (%)**			
		>500,000	1 (100.0)	
	**Education, n (%)**			
		University degree or higher	1 (100.0)	
	**Income, n (%)**			
		>$100,000	1 (100.0)	
	**Employment status, n (%)**			
		Retired	1 (100.0)	
	**Marital status, n (%)**			
		Married or common law	1 (100.0)	
	**Chronic condition, n (%)**			
		Chronic kidney disease	1 (100.0)	
		Diabetes	1 (100.0)	
		Other	1 (100.0)	
	**eHEALS score**			35 (undef^d^)
	**SUS total**			5 (undef)

^a^All dollars are in CAD. Study data were mostly gathered in Summer 2023 and so a currency conversion rate from August 16, 2023 (CAD/1.3518455345177844/0.7397294842244747) is used.

^b^eHEALS: eHealth Literacy Scale.

^c^SUS: System Usability Scale.

^d^Undef: undefined.

**Table 4:** Usability testing results.

Issue			Recommendation
**Navigation and workflow**			
	Page 4 of 5 (onboarding) does not start at the top of the page: Page 4/5 during onboarding loads with the middle section displayed with no scroll bar.	Ensure that all pages load correctly.	
	Missing “Next” button leads to unexpected and surprising app behavior: Some pages have “next” buttons and others do not.	Ensure that all pages with steps in a workflow include the “Next” button.	
	Ensure that notifications do not go to junk mail: Users noted from previous experiences that notification emails sometimes end up in their junk folder, but they may not always think to check there.	After onboarding, a notification should appear prompting users to check their junk folder if they have not received the email.	
	Fear that a clinician would not discuss information over the phone: Many users lack confidence that their HCP^a^ will offer advice over the phone and are unsure about the type of guidance they will receive. The example was specific to insulin adjustments.	If users are advised to call their HCPs in any situation, the app advises them that this type of guidance can be given over the phone. HCPs should also remind patients that HCPs can communicate dosage changes over the phone.	
	Users want to take proactive steps to prepare for sick days: Users find the self-management advice helpful but can access this advice only during a sick day event. They would like to proactively prepare for a sick day.	Enhance the resource page by emphasizing self-management tips for mild to moderate symptoms. Make archived advice more noticeable.	
	Users want their HCP aware of sick days and advice provided: Users are concerned that their HCPs will be unaware of a sick day occurrence and what advice was given.	The research team will review this issue, as general practitioners expressed reluctance to interact with notifications from the app and follow up with their patients.	
**Layout and presentation**			
	Text alignment and item grouping vary: The alignment and configuration of the conditions and topics during the onboarding process impede users from easily scanning the options.	Organize the conditions either alphabetically or by their relatedness. In addition, ensure that the lists are left-justified and convert the items into checkboxes instead of buttons.	
	Layout and design present issues on small phones: Users with small phones could not see the “next” button or text below the “fold.” Screens have no indication to scroll.	Add a scroll bar to the side of the screen to indicate that scrolling is required. Ensure that key elements are at least partially visible on the “fold.”	
	Ensure responsiveness to changing aspect ratio: When users turned their phone to landscape, potential layout issues arose. The prototype was not able to respond to orientation build, so this was identified as something to monitor during development.	Ensure that the app is responsive to changing device orientation, and the layout is usable and esthetically sound for all orientations.	
	Narrow portrait screen makes options difficult to read: Some content is difficult to read in a narrow portrait layout, especially when lists are not organized in descending order.	Ensure that text is succinct and left-justify lists with 1 item per row to support scanning.	
	Include medication doses and photographs in the medication list: Users expressed challenges in identifying medications solely by their names and raised concerns regarding the absence of dosage information.	Add photographs of medications and ensure that the medication list contains details regarding doses and frequency.	
**Visual elements**			
	Icon clarity: Icons used within the prototype were not recognizable by several users.	Use icons sparingly, and icons used should be clear and adhere to WCAG^b^ 2.1 accessibility guidelines [43]. To promote a better understanding of functionality, use text instead of icons (eg, “edit” and “add”).	
	Ensure sufficient contrast for text colors: Some text was very light in color and difficult to read.	Ensure sufficient contrast between background and text using the WCAG 2.1 accessibility guidelines.	
	Use colors and shapes consistently: In certain instances, a dark blue circle served as an indicator for a clickable element, while on other pages, it is used purely for decorative purposes.	Maintain consistent color usage throughout the app to prevent any possible confusion. Refrain from applying buttons or link colors to elements that are not interactive.	
	Use red sparingly and intentionally: Users assume that red button colors indicate an alert, danger, or stop. Users also find that button colors are inconsistent.	Use red sparingly and intentionally and reserve it for error messages. Ensure that button colors are applied consistently and match the context.	
**Functionality and user experience**			
	Face and fingerprint recognition may be insufficient: Users shared their personal experience with apps and facial recognition not working if they wear glasses. Some users were unsure how to use fingerprint recognition.	Ensure multiple ways to log on to the app and consider removing face and fingerprint recognition altogether.	
	Concerns around the workload of manual medication entry: Users raised concerns about the process of inputting their medications manually, such as errors and the amount of time it would require.	Have the app retrieve the list of medications from relevant pharmacies, NetCare, or an alternative platform.	
**Interactivity**			
	App lacks a “Select all” option: When a list contains numerous options, users prefer the ability to “select all” items at once.	Add a “Select all” option to lists where appropriate.	
	Comfort with a computer versus a smartphone: Certain users prefer using smartphones for calls and texts and are uncomfortable with touch screens. These users would prefer to work on a computer.	If possible, provide a desktop or web version of the app for users who prefer this option.	
	Lack of checkboxes for selection: Users find it unclear whether they have selected options when only a color change indicates selection.	Make options into a checklist.	
	“X” button does not always function: The “X” close button did not always work.	Ensure that interactive elements (such as the “X” button) work as expected.	
**Medical terminology**			
	Typos in resources (Diabetes Canada handout): Users have identified typos and formatting issues in the Diabetes Canada handout.	Ensure that all content, resources, and handouts are meticulously checked for typos, grammatical errors, and formatting issues, regardless of their source.	
	Users forget which blood pressure number is systolic and diastolic: Some users were unsure which blood pressure number is systolic (top) and which is diastolic (bottom). In addition, the units of measurement (mm Hg) can be confusing.	Include “top number” or “bottom number” in brackets whenever the tool mentions diastolic or systolic. Ensure that this is consistent throughout the app. Consider removing units to avoid confusion, still to be discussed.	
	Users are unsure what NSAIDs^c^ are: Users are unsure what an NSAID is, even when provided with examples of the generic and brand names.	Remove the term “NSAID” or use statements such as “Do you take any of these over-the-counter medications (nonsteroidal anti-inflammatory drugs)?”	
	Users are unsure what ketones are: Some users are unfamiliar with ketones, as they do not track this information. This situation can arise during onboarding if users accidentally select an option they do not monitor at home.	Consider implementing a confirmation page during onboarding to verify that the selection choices accurately reflect what users monitor at home.	
**SDMG^d^** **content**			
	Unsure what “cope with symptoms” means: Some users find the question “Can you cope with managing these symptoms on your own?” difficult to interpret. The term “coping” is complex and vague.	Rephrase the question to be more specific: “Are you able to manage these symptoms by yourself?”	
	The context of “feeling better” and “feeling worse” in relation to living with chronic diseases is unclear: These phrases are subjective and context-sensitive, particularly for individuals living with chronic diseases, who may never feel “better” compared with their predisease “normal.”	Ensure language clarity and avoid vague and ambiguous statements that can be misinterpreted.	
	Users are confused and startled when told to go to urgent care or call 911: Users felt that they would not be able to comfortably explain to the 811/911 operator or the urgent care doctor why they were calling or at urgent care.	At the beginning of the app, inform users that they may receive advice to go to urgent care or call 811/911 based on symptoms. Provide clear and detailed explanations about why users are being asked to do this. Add a button “Information for Healthcare Providers” on the advice page so that users can share information with the emergency doctor.	
	Type of pill bottle not specified in medication list: Some users felt that it would be beneficial to have the type of pill bottle listed as they have issues opening the standard type.	Include additional information on the type of pill bottle a medication is dispensed in to ensure that all relevant details are accurately captured.	
	Limited resource topic areas: Most resources are tailored for people with diabetes and not other conditions. This may lead users to question whether they are the intended audience.	Include resources and information for all the conditions the app addresses. Ensure that the app’s splash screen communicates the intended audience.	
	Limited medical conditions: Users felt that the guidance was based on conditions rather than medications. They were concerned that their symptoms might be related to a condition or topic not covered.	Consider including additional chronic conditions. Clarify that the focus is on medications, not necessarily specific conditions.	
	Difficult to answer medication frequency question: Users found it challenging to answer the refill frequency question; not all medications are refilled at the same time.	Remove this question.	
	Are resources for SDMG? Users were unsure whether resources were specific to SDMG, their condition or health information in general.	Label the resources to indicate whether they pertain specifically to SDMG or are general health information. Consider removing all non-SDMG resources.	
	Context needed for what each symptom entails: Users have reported that some symptom-related questions reflect their usual condition experiences, leading to confusion. For example, people with blood pressure issues often get lightheaded and people with hypertension can have blood pressure fluctuations as part of their condition.	Clarify that these questions are intended to identify symptoms that are sudden in onset or significantly different from the user’s normal experience.	
	Blood pressure measurement questions assume that users have a home device: Users have noted that the questions about blood pressure seem to assume that they can measure their blood pressure at home.	Adjust the question to include, “If you have a blood pressure device at home.” In addition, provide an “N/A” option.	
**Content**			
	Users misunderstood the term “communication preference”: Some users thought that the term “communication preference” was for language preference (eg, French or English) rather than the method (eg, text or email).	Replace “communication preference” with “notifications” in the user profile. List the options as “email,” “text,” and “email and text.” During onboarding, remove the term “communication preference” and replace it with, “Please let us know how you prefer to be notified.”	
	Users need reminders of their previous symptoms: Users have noted that recalling previous symptoms can be challenging, which makes it difficult to answer questions or interact with the app effectively.	Include a summary of the user’s last check-in or a history of recent symptoms.	
	Users forgot the purpose of the app: The app lacks introductory information to explain its purpose and what users can expect. Users mentioned that they would use the app only when unwell, which could lead to forgetting its purpose due to infrequent use.	Provide clear and concise information about the app’s purpose, target audience, and expected benefits. Offer optional guided tour screens that demonstrate how to use the app, include a brief video and a handout with this information, and provide detailed information on conditions and medications that fall under SDMGs.	
	Issue with language and terminology used: Some users were unaware of what caffeine is and did not realize that coffee and other drinks contain it.	Add examples in brackets to ensure that users understand what is being referred to. For instance, “coffee (or tea, coke, or instant flavored coffee) contains caffeine.” Provide clear definitions and examples to enhance clarity and understanding.	
**Target audience**			
	Users with vision issues had difficulty using the app: Several users found the text small, which could be problematic for users with vision issues, a common concern for the older adults and people living with diabetes.	Increase the text size and provide options for text resizing within the app. Consider implementing a high-contrast mode to further enhance readability. Ensure W3C Content Accessibility Guidelines (WCAG 2.1) are adhered to.	
	Users have concerns regarding data security and privacy: Users misunderstand the implications of downloading and using an app, believing that avoiding online platforms means their data are not stored. Some users expressed concerns about their privacy and how the research team will handle their data.	Provide clear information about how data are stored and managed, emphasizing that data are already stored digitally even if users avoid engaging with online platforms. Include a section in the app that transparently outlines the privacy policy and data usage terms.	
	Some users use more than 1 pharmacy: Some users use different pharmacies for various medications and are concerned about whether medications obtained from non-PC pharmacies fall under SDMG and how that would be managed.	Ensure that app resources have sections that clearly explain which medications fall under SDMG, regardless of the pharmacy they are obtained from. Let users know that as of now the app is only available for PC Health app users, but SDMG still applies.	
	Lack of real-life practical language throughout the app: Users were unclear about the app’s purpose, the meaning of “sick day medication guidance,” and the phrase “feel worse than my normal.”	Revise the app’s language to be more accessible to nonmedical users. Avoid medical jargon, use plain language, and provide definitions and use examples.	
**Branding**			
	Pause logo was found to be confusing: Users did not recognize the logo as a pill	This was not an issue for all users, so the logo could be left as is or consider redesigning the logo.	

^a^HCP: health care provider.

^b^WCAG: Web Content Accessibility Guidelines.

^c^NSAIDs: nonsteroidal anti-inflammatory drugs.

^d^SDMG: sick day medication guidance.

### The PAUSE App UI Design

#### Overview

**Figure 2:**
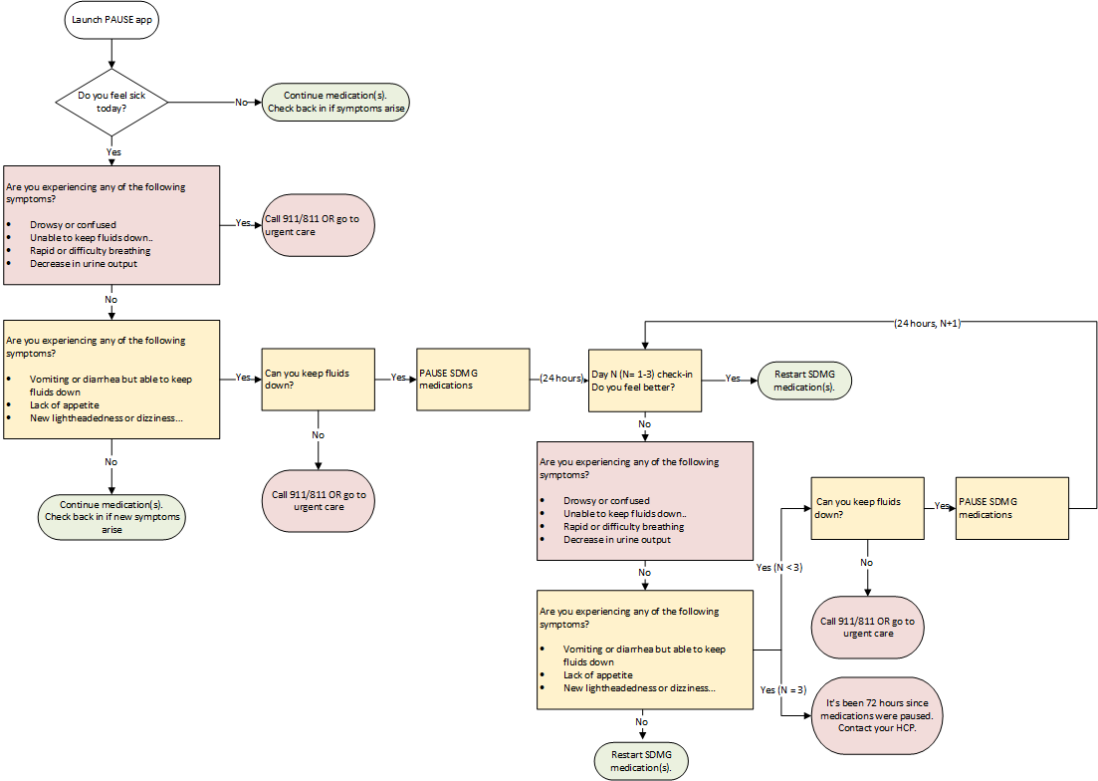
PAUSE app flow showing how different patient responses result in pausing or resuming medications or seeking urgent care for serious symptoms. Yellow indicates mild symptoms that can be managed at home by pausing specific SADMANS (sulfonylureas, angiotensin-converting enzyme inhibitors, diuretics, metformin, angiotensin receptor blockers, nonsteroidal anti-inflammatory drugs, and sodium-glucose cotransporter 2 inhibitors) medications. Red indicates situations where urgent care should be sought. Green indicates that the patient has no more risky symptoms and can resume their medications. HCP: health care provider; PAUSE: Preventing medication complications during AcUte illness through Symptom Evaluation and sick day guidance; SDMG: sick day medication guidance.

Figure 2 shows the overall flow of content within the app and user experience for the most current prototype of the PAUSE app.

#### Sick Day Medication Guidance

**Figure 3:**
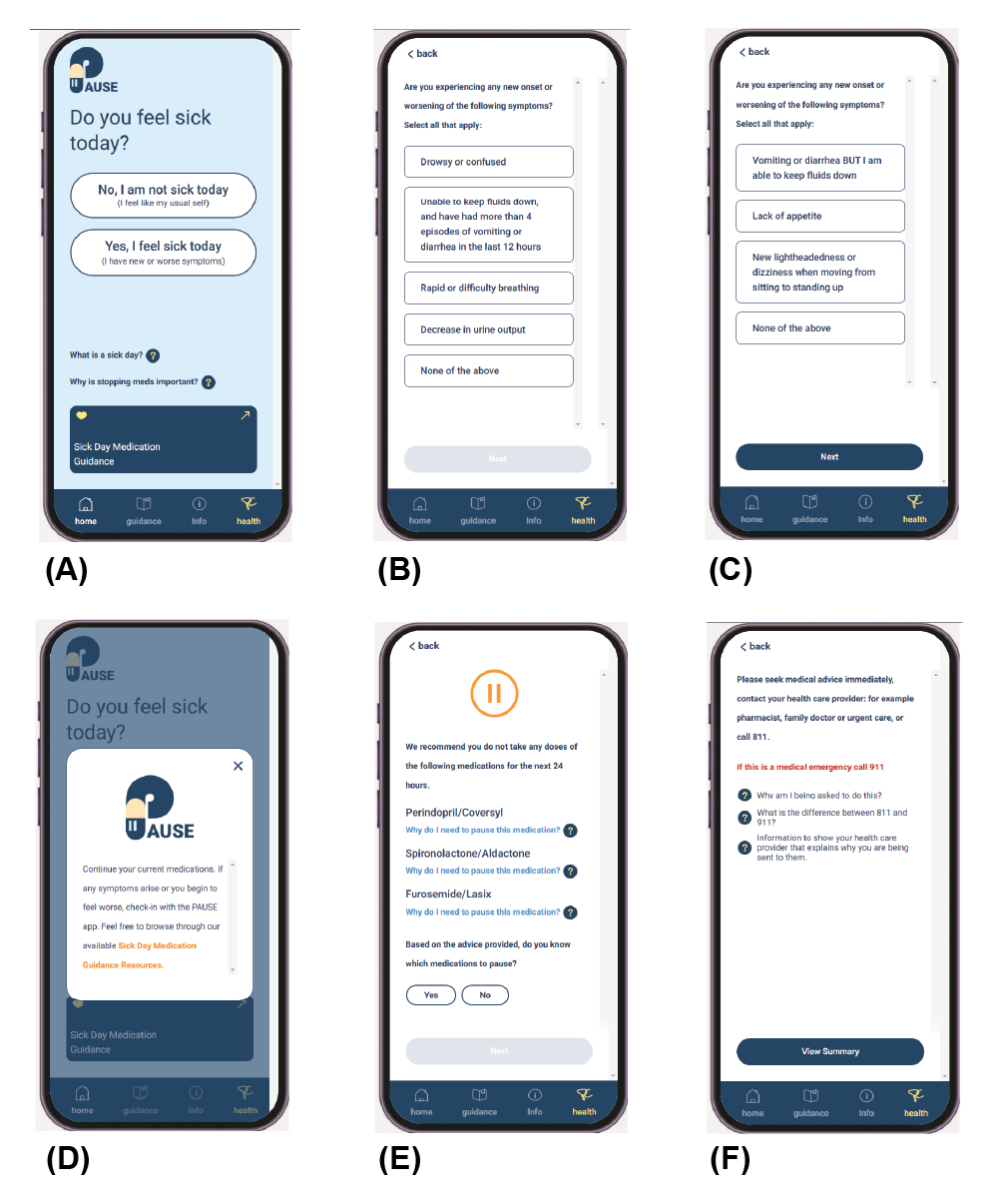
Screen captures of the prototype Preventing medication complications during AcUte illness through Symptom Evaluation and sick day guidance (PAUSE) app UI at key moments in sick day medication guidance. (A) The app’s initial screen shown when launching the app ahead of activating an acute illness tracking and support flow. (B) First screening of symptoms if the user has indicated that they are feeling unwell. Responses to this screen trigger other screens, as shown in the app’s flow diagram. (C) Second layer of symptom screening if the patient has indicated that they have any of the previous screen symptoms. (D) Advice provided if the symptoms are deemed mild. (E) Advice to pause specific medications (based on the patient’s list of medications) in response to moderate symptoms. (F) Advice to seek urgent care immediately as a result of patient-reported severe symptoms.

The PAUSE app UI prototype addresses SDMG in a personalized and time-sensitive manner. The app guides the patient through assessing the severity of their symptoms, appraising their risk of volume depletion, and recommends any temporary medication adjustments to be made. Through push notifications, the app follows up with the patient to reassess symptoms and provide additional recommendations or adjustments. If patient-reported symptoms are severe enough, the app will also recommend that urgent or emergency care be sought. Prompts for resuming medications are provided once qualifying sick day symptoms have resolved. Figure 3 shows key SDMG moments in the PAUSE prototype app UI, including (A) the initial check to see whether the user is feeling unwell, (B) serious and subsequent, and (C) less severe symptoms to be assessed, followed by advice for the user to follow under (D) mild, (E) moderate, and (F) urgent symptom presentation situations.

The prototype PAUSE app UI assumes that the app will be integrated with an existing patient health record and active medications list; the app will be able to automatically flag qualifying medications that have been prescribed to the patient to be carefully adjusted or temporarily paused to better support the patient during an acute illness.

#### Onboarding

The PAUSE app’s user flow designs support the diverse needs of patients (ie, patients with chronic conditions needing SDMG and their caregivers), providing tailored experiences for different groups, including individuals with no diabetes, and those managing diabetes with or without insulin therapy. As referenced in Figure 2, the onboarding user flow gathers information needed to support appropriate and personalized sick day medication adjustments to deliver customized care [44].

#### Check-in

The check-in user flow is operationalized via push notifications in the days following the patient’s initial reporting of symptoms constituting a “sick day” on the app. The prompts check on key symptoms and provide recommendations for how to manage the ongoing acute illness. Figure 2 shows potential outcomes: (1) mild symptoms: continue medication; (2) moderate symptoms: adjust insulin doses and pause specific medications from the SADMANS list, as well as general health advice such as staying hydrated and providing some guidance on hydration; and (3) severe symptoms: seek medical attention urgently.

If the app prompts the patient to seek medical attention, this prompt includes a summary of the information that they are encouraged to share with medical practitioners to support potential symptomatic cognitive impairment, patient knowledge, and awareness, and to promote transparency and trust-building around *why* the app recommends that they urgently seek medical attention.

#### Other Support Materials

The app also includes additional materials and resources. The home screen’s bottom navigation bar is visible throughout the app to help the user be aware of the section. Currently, this section includes the Diabetes Canada SDMG handout to ensure that users would find the materials easily [45]. As the PAUSE prototype is developed into a functional platform, additional materials and guidance can be added to this section.

## Discussion

### Principal Results

This work showcases the iterative creation and evaluation of an SDMG innovation we previously identified as a key gap in support for SDMG knowledge, awareness, and patient self-management [11]. Through this work, we demonstrate the value of HCD approaches, specifically heuristic evaluation and usability testing, to design a digital SDMG self-management tool that is suited to the specific needs and context of patients requiring SDMG. We designed the resultant PAUSE app UI prototype to support patients to identify and appropriately adjust their medications on sick days.

### Comparison With Prior Work

SDMG requires novel solutions for implementation and further evaluation. A study by researchers in Australia found that uptake of SDMG recommendations was poor and that pharmacists recommended laboratory monitoring for potential toxicity but did not provide SDMG despite 71.1% of community-dwelling patient participants using at least one of the SADMANS medications and Kidney Health Australia recommending temporary cessation of these medications during episodes of acute illness for patients with CKD [46]. They concluded that more research is needed to understand barriers to providing SDMG to patients. Another study conducted in The Netherlands found that for both patients with and with no impaired kidney function, most patients continued to take their prescription drugs without adjustment during sick days [47]. This work also noted the challenge created by separating information provided to patients in medical appointments (encouraging consistent adherence) from sick day situations, which happen unexpectedly, and the proper information may not be readily at hand.

SDMG digital tools are an innovation to address sick day needs of patients with diabetes and kidney and cardiovascular conditions who use specific medications that could be harmful during acute illness. Prior research suggests that a digital tool might be an acceptable and feasible strategy to help patients self-manage SADMANS medications during acute illness [48]. Notably, a recent scoping review on sick day management in people with CKD found that a digital intervention would likely be easy to use and an acceptable approach to providing SDMG [16]. Our team previously conducted focus groups and found that both patients and health care practitioner participants supported the concept of a digital tool for SDMG [12]. We have also explored barriers and enablers for both patients and HCPs through focus groups and identified built-in prompts for prescribing and dispensing software as one potential solution [12]. The COVID-19 pandemic also catapulted technology into the role of patient-provider intermediary [17,49]. One review identified strategies for improving kidney disease care using technology, including remote patient monitoring, patient education, and health-related apps to support self-management [50].

This current design and evaluation work builds upon our team’s previous work and pairs it with key elements of HCD, namely, heuristic evaluation and usability testing conducted iteratively on successive versions of the PAUSE app UI prototype. A similar SDMG intervention, the Sick Day Protocol developed by the National Health Service Highland in Scotland to prevent acute kidney injury, was iteratively designed, tested, and refined using similar approaches. Patients with sick day medications were given a business card with drugs considered “hazardous” for sick days and instructions to stop those medications for 48 hours while sick, as well as a pamphlet and (as part of a clinical trial) a telecommunications platform to report and monitor sick day events [15]. Usability testing was conducted on the final protocol and card versions and revealed that patients were generally unable to identify which medications should be withheld during a sick day scenario [51]. The lack of patient involvement in earlier phases of the protocol and card design resulted in delayed issue identification, halting project progress after significant resource investment. Informed by this prior research knowledge, we followed a comprehensive HCD approach including heuristic evaluations and usability testing on predevelopment prototypes of the PAUSE app, ensuring a usable design and content, and that patients can act on the app’s recommendations prior to final design and development. The app was conceived and iteratively designed using an HCD approach, considering likely users (ie, the patients), lack of recall of information from a routine physician check-in, and their potentially impaired cognitive states during applicable sick days. In creating the PAUSE app prototype, the end user, the patient, was at the center of all design efforts and the app’s interface and information flow designed accordingly.

### Limitations

This work is not without limitations. Our heuristics evaluations and usability testing analyses were rapid, with the goal of providing timely feedback to the development team for integration into subsequent prototype versions. As such, the analysis approach was practical and pragmatic oriented, so some nuances related to reported elements and issues may have been missed. As the key value of heuristic evaluations and usability testing is in the practical application to real-world software development projects, nuances are less important, justifying the approach taken. The prototype UI is in English and the participants who completed the usability testing spoke and read English; however, we recognize that future iterations and integrations will need to consider other languages to be universally useful. We had 6 individuals partake in the usability testing; while this sample may be interpreted as small, it is in accordance with formative usability testing guidelines where approximately 80% of the issues are identified, which is intended as a happy balance between effort to solicit feedback and value-add of additional participants [35,36].

The app is being designed to function with minimal training, which supports real-world usability but may limit its usability among populations with lower digital literacy or limited access to mobile technology. This is a key question facing many digital health interventions, where those with access to technology can benefit but those without access are left behind. Reducing barriers to technology access might be one way to address this “digital divide” but is beyond the scope of this current work.

### Conclusions

Within an HCD approach, we designed and refined an SDMG self-management prototype app for patients with diabetes, cardiovascular disease, and CKD. Heuristics evaluation and usability testing conducted iteratively between prototype revisions led to a final prototype design with key user flows and integrated supports for acting on severe acute illness situations where pausing SADMANS medications is recommended. The refined PAUSE app is well positioned to better support patients and their caregivers who are on specific medications for diabetes, cardiovascular disease, and CKD to more effectively self-manage at home and in accordance with current guideline recommendations. The HCD approaches used allowed us to address technical and workflow barriers using iterative heuristics evaluation and usability testing of the PAUSE app prototype design; however, we recognize that there remains an impetus for patients to remember to initiate the PAUSE app during an acute illness—which was out of scope of the present evaluation. Further research is needed to implement and evaluate the PAUSE app in community-based care and ensure that patients can accurately follow SDMG using the app, especially in times of acute illness.

## Appendix

Multimedia Appendix 1 Usability testing interview guide.

Multimedia Appendix 2 Preventing medication complications during AcUte illness through Symptom Evaluation and sick day guidance heuristic evaluation results overview, issues within a category shown as a percentage of the total number (n=21). SDMG: Sick day medication guidance.

Multimedia Appendix 3 Heuristic evaluation results.

Multimedia Appendix 4 Usability testing results overview. Each category is shown as a percentage of the total number (n=44) of issues identified. SDMG: sick day medication guidance.

Multimedia Appendix 5 Usability testing results.

## Abbreviations

CKD: chronic kidney disease
eHEALS: eHealth Literacy Scale
EMR: electronic medical record
HCD: human-centered design
HCP: health care provider
PAUSE: Preventing medication complications during AcUte illness through Symptom Evaluation and sick day guidance
SDMG: sick day medication guidance
UI: user interface
