# Supplementary material for: Using Human-Centered Design and Development to Create a Digital Sick Day Medication Guidance Application for People With Diabetes, Cardiovascular Disease, or Chronic Kidney Disease: Mixed Methods Study
Source: JMIR Form Res. 2025 Nov 27;9:e77240. doi: 10.2196/77240 (PMC12778901; doi:10.2196/77240)
Supplement: Multimedia Appendix 1 [file formative_v9i1e77240_app1.docx]

## Multimedia Appendix 1: Usability testing interview guide.

### Part 1: Demographic Questionnaire (Sent in advance via email link to RedCAP Survey)

- Before we start working with the app itself, I’d like to ask you some questions about yourself. Your answers to these questions will help us understand better who we have talked to and will let us summarize that information when we report the findings of this study.

1. **How do you describe yourself?**

- Patient
- Caregiver

1. **If you are a patient check off all the conditions that you are living with?**

- Chronic kidney disease
- Heart failure
- Diabetes
- Other_

1. **If you are a care giver, please check off the conditions the person you care for has,**

- Chronic kidney disease
- Heart failure
- Diabetes
- Other_

1. **What is your age?**

- 20-40 years
- 41-50 years
- 51-60 years
- 61-70 years
- Over 70 years
- I prefer not to answer

1. **What is the size of city/town where you live?**

- Population greater than 500,000
- Population between 100,000 and 500,000
- Population between 10,000 and 99,000
- Population less than 10,000
- Other or uncertain
- I prefer not to answer

1. **What is your highest level of education?**

- Grade 12 or less
- College or technical certificate / diploma
- University degree
- Graduate degree
- Other:
- I prefer not to answer

1. **What is your annual household income?**

- $0 - $29,999
- $30,000 - $49,999
- $60,000-$69,999
- $70,000-$99,999
- $100,000 - $149,999
- $150,000 or more
- I prefer not to answer

1. **What is your current employment status?**

- Full-time
- Part time
- Casual
- Home-duties
- On disability
- Retired
- Student
- Not employed
- Other:
- Prefer not to answer

1. **What is your marital status?**

- Married
- Common-law
- Widowed
- Single
- Separated or Divorced
- I prefer not to answer

1. **What is your sex? (i.e., your biological identity on your birth certificate)**

- Male
- Female
- Intersex
- Prefer not to answer

1. **What best describes your gender identity?**

- Cisgender man
- Cisgender woman
- Transgender man
- Transgender woman
- Non-binary person
- Gender-fluid
- Gender-queer
- Gender non-conforming
- Demi-gender
- Agender
- Two Spirit
- I prefer to self-identify as:
- Prefer not to answer

1. **How do you primarily identify? (Select all that apply)**

- First Nations, Metis, Inuit, or other North American Indigenous
- Arab
- Asian-East (e.g., Chinese, Korean, Japanese)
- Asian-South-Ease (e.g., Filipino, Vietnamese)
- Asian-South or Indo-Caribbean (e.g., Indian, Pakistani, Guyanese)
- Asian-West (e.g., Iranian, Afghan)
- Black-Canadian/American
- Black-African (e.g., Ghanaian, Ethiopian, Nigerian)
- Black-Afro-Caribbean or Afro-Latinx (e.g., Jamaican, Haitian, Afro-Brazilian)
- Latin American (e.g., Brazilian, Mexican, Chilean, Cuban)
- White (e.g., European, French, Ukrainian, Euro-Latinx)
- I prefer to self-identify as:
- Prefer not to answer

### Part 2: eHealth literacy Scale (eHeals) (Sent in advance via email link to RedCAP Survey)

- Thank you so much for answering those questions for us. The next few questions ask you for your opinion and about your experience using the Internet for health information. For each statement, tell me which response best reflects your opinion and experience right now.

1. How **useful** do you feel the Internet is in helping you in making decisions about your health?

| 1 | 2 | 3 | 4 | 5 |
| --- | --- | --- | --- | --- |
| Not useful at all | Not useful | Unsure | Useful | Very Useful |

1. How **important** is it for you to be able to access health resources on the Internet?

| 1 | 2 | 3 | 4 | 5 |
| --- | --- | --- | --- | --- |
| Not important at all | Not important | Unsure | Important | Very important |

1. I know **what** health resources are available on the Internet
2. Strongly Disagree
3. Disagree
4. Undecided
5. Agree
6. Strongly Agree
7. I know **where** to find helpful health resources on the Internet
8. Strongly Disagree
9. Disagree
10. Undecided
11. Agree
12. Strongly Agree
13. I know **how** to find helpful health resources on the Internet
14. Strongly Disagree
15. Disagree
16. Undecided
17. Agree
18. Strongly Agree
19. I know **how to use** the Internet to answer my questions about health
20. Strongly Disagree
21. Disagree
22. Undecided
23. Agree
24. Strongly Agree
25. I know how to use the health information I find on the Internet to help me
26. Strongly Disagree
27. Disagree
28. Undecided
29. Agree
30. Strongly Agree
31. I have the skills I need to evaluate the health resources I find on the Internet
32. Strongly Disagree
33. Disagree
34. Undecided
35. Agree
36. Strongly Agree
37. I can tell **high quality** health resources from **low quality** health resources on the Internet
38. Strongly Disagree
39. Disagree
40. Undecided
41. Agree
42. Strongly Agree
43. I feel **confident** in using information from the Internet to make health decisions
44. Strongly Disagree
45. Disagree
46. Undecided
47. Agree
48. Strongly Agree

***Thank you!***

** Note: Questions #1 and #2 are recommended as supplementary items for use with the eHEALS to understand consumer’s interest in using eHealth in general. These items are not a formal part of the eHealth Literacy scale, which comprises questions #3-10.*

### Part 3: Introduction

- Hi, <participant’s name>. Thank you for agreeing to help us evaluate a new App called the PAUSE APP, which is a decision support tool to help you better manage your medications when you are sick. My name is <facilitator’s name>, and I’m going to be walking you through this session today as well as taking some notes.
- Before we begin, I have some information for you, and I’m going to read it to make sure that I cover everything. You probably already have a good idea of why we asked you here but let me go over it again briefly. We’re asking people who have a non-communicable disease, such as diabetes, high blood pressure, and chronic kidney disease and take medications for these diseases to try using a sick-day medication guidance app that we’re working on. The session should take about 1 hour of your time.
- The first thing I want to make clear right away is that we’re testing the app, not you. You can’t do anything wrong here. In fact, this is probably the one place today where you don’t have to worry about making mistakes.
- Today we’re working with a prototype, not the real app. It looks similar to what the real app will look like, but there’s no brains behind what you see on the screen, and so there will be things that don’t “work”. It’s kind of like crossing a creek by stepping on stones to stay out of the water. Don’t worry though, we’re here to help you get from one stone to the next.
- The prototype is also a bit delicate – things will be slow to load, and the prototype tool will get confused if we try to click on too many things too quickly. Things work as long as we’re patient, and we will help you out as we go.
- As you use the prototype, I’m going to ask you to think out loud as much as possible: to say what you’re looking at, what you’re trying to do, and what you’re thinking. This will be a big help to us. Once you are finished working through the scenarios, I will ask you questions about your experience with the system.
- Feel free to ask me questions. I may not be able to answer them right away, since we’re interested in how people do when they don’t have someone sitting next to them to help. But if you still have any questions when we’re done, I’ll try to answer them then. And if you need to take a break at any point, just let me know.
- (IN PERSON) You may have noticed the audio recorder. With your permission, I’m going to record our session. We will only be recording your voice. The recording will only be used to help us figure out how to improve the app, and it will only be reviewed by the people working on this project. Any information that we record or that you provide will remain confidential and you will not be identified when we share the results of the study.
- (VIRTUAL) We’re going to ask you to share your screen so we can follow along with what’s going on. With your permission, we will be recording our Zoom session but will only be using the audio recordings as part of our data collection. Zoom will also be collecting a video recording (a setting we can’t change for video recordings we create on a local computer), but once the session is complete, we will be deleting the video recording everywhere. The audio recording will only be used to help us figure out how to improve the app, and it will only be reviewed by the people working on this project. Any information that we record or that you provide will remain confidential and you will not be identified when we share the results of the study.
- If you would, I’m going to ask you to review this Consent Form and sign the last page for us.

**Provide participant with the Information Letter and Consent Form, give them a few minutes to review and sign.**

Do you have any questions for me before we begin?

### Part 4: Your experience and familiarity with using mobile applications or digital tools

- I have a few questions about your previous experience and familiarity with using mobile applications or digital tools. Again, there’s no right or right answer here, we’re mostly looking to learn more about the people we’re hoping to help.

1. Tell us a little bit about your experience with using a computer? Smart phone, etc…
2. Tell us about your experience with using any mobile apps or websites for caregiving or health management?
3. (If applicable) What are some of the main tasks you typically perform using caregiving or health management apps?

**Probes (if participant needs more concrete guidance to answer)**

- What are the key challenges or pain points you face when using caregiving or health management apps?
- Are you currently using any caregiving or health management apps?
  - 1. If yes, please mention the names and briefly describe your experience with them.
- How often do you use mobile applications or software related to caregiving or health management?
  - 1. Hints: Apple Health, Garmin, Fitbit, PC Health, Telus Health, BrightSquid
    2. Hints: Glucose control or blood pressure in an app…

### Part 5: Prototype overview

- **Have participants open the prototype link on their preferred device.**
- First, I am going to ask you to look at the prototype, and tell me what you think about it, what strikes you about it and what do you think it is for. We’re not going to click on anything just yet, but you can use your mouse or finger to move the cursor around the screen.
- **This should only last about 2-3 minutes**
- **Notes:**

### Part 6: (Sample) Scenarios and Tasks

- Thank you. Now I’m going to ask you to work through a scenario with a few tasks. I’m going to read it out loud and give you a printed copy. I am going to ask you to pretend to be this individual and go through the app as you think they should.
- **Participant will be asked to complete Scenario A and ONE OF B, C, or D depending on the best alignment of the persona with their own personal situation.**

#### SCENARIO A (Onboarding, feeling normal)

Persona: Ariel Dorset

DOB: 25 May 1963

- Communication preferences = text messages only
- Topics interested in = diabetes, high blood pressure, and diet
- Monitoring = yes to all of them
- Currently receive your pills = pill bottle
- Fill them = every 3 months
- Do you take any of these over-the-counter medications = yes (rarely)
- Pharmacy = Shoppers Drug Mart #2420, 120 Fifth Ave Cochrane.

##### Task 1: You are accessing the App for the first time. Please create an account using the above information.

- **Once the patient is able to set up a profile, begin the tasks:**

##### Task 2: You are feeling normal (or well today) and want to see how the app functions, please sign into the app. Where would you go next, and what would you do?

##### Task 3: You are wanting to review the Diabetes Canada sick day handout. Please navigate to the resources and select the handout.

##### Task 4: You are wanting to review your medication list and profile to make sure they are accurate. Please navigate to these two pages.

#### SCENARIO B (non-diabetes flow)

***Persona details***

Ariel Dorset

DOB: 25 May 1963

You have previously created an App.

Medications you are taking:

- perindopril/Coversyl
- spironolactone/Aldactone
- atorvastatin/Lipitor
- metoprolol/Betaloc
- furosemide/Lasix

##### Task 1: You have been up overnight vomiting but able to keep water and juice down. You are about to take your morning medications and want to check the advice in the app first.

- You have taken your blood pressure and it is 134/75 mmHg (it’s usually 146/82 mmHg).
- You have no other home monitoring readings.
- You are able to cope with these symptoms.
- You are able to drink, but haven’t tried keeping any food down yet.

***Use the app as though you were experiencing these symptoms. How would you use it?***

##### Task 2: It is now 24 hours after you first vomited and you now feel worse. You can’t keep fluids down and the app has sent you a notification for follow up. Please use the app to check in.

##### Task 3: It’s a few weeks later and you wake up one morning and have severe diarrhea and can’t seem to keep anything down. Sign into the app and use it as though you were seeking medical advice.

#### SCENARIO C (diabetes flow, no insulin)

***Persona details***

Ariel Dorset

DOB: 25 May 1963

You have previously created an App.

Medications you are taking:

- perindopril/Coversyl
- spironolactone/Aldactone
- atorvastatin/Lipitor
- metformin/Glucophage
- empagliflozin/Jardiance
- glyburide/Glyburide (Sanis)

##### Task 1: It’s the weekend, you have no appetite and are feeling lightheaded, You are about to take your evening medications and want to check the advice in the app first.

- You have had normal blood glucose readings throughout the day.
- You have no other home readings.
- You are feeling ok with managing your symptoms.
- You can eat and drink but don’t feel like food.

##### Please use the app as if you were experiencing these symptoms.

- **(Start participants on the follow-up screen)**

##### Task 2: It is now 24 hours after you first said you were lightheaded and had no appetite, and the app has sent you a notification for follow-up. You have started getting some low blood glucose readings with 5 low readings throughout the day. What do you do?

##### Task 3: It is now 48 hours after you first said you felt lightheaded and had no appetite. You are now feeling better, and the app has sent you a notification for a follow up. What do you do?

#### SCENARIO D (diabetes, insulin)

***Persona details***

Ariel Dorset

DOB: 25 May 1963

You have previously created an account on the App.

Medications you are taking:

- perindopril/Coversyl
- spironolactone/Aldactone
- atorvastatin/Lipitor
- insulin glargine (long-acting basal insulin)
- insulin aspart (rapid-acting bolus insulin)

##### Task 1: It’s the weekend, you have no appetite with a decrease in how much water you are drinking. You are about to take your morning medications and want to check the advice in the app first.

- You have checked your ketones using a urine ketostix and it showed negative for the presence of ketones.
- Your blood glucose readings are coming back normal.
- You have no other home monitoring readings.
- You are able to cope with these symptoms.
- You are able to drink, but haven’t tried keeping any food down yet.

***Please Use the app as if you were experiencing these symptoms.***

##### Task 2: It is now 24 hours after you first said you felt lightheaded and had no appetite. The app has sent you a notification for follow up. You have tested your ketones again and the urine ketostix shows small presence of ketones. What do you do?

##### Task 3: It is now 48 hours after you first said you felt lightheaded and had no appetite. You are now feeling better. The app has sent you a notification for follow up. What do you do?

- **At this point have the note taker fill in the Incident Report Log as participant works through the tasks.**
- **Allow the participant to proceed until you don’t feel like it’s producing any value or the user becomes very frustrated.**
- **Continue until time runs out.**

### Part 7: POST-EVALUATION QUESTIONNAIRE (for all participants)

- First, I want to take a moment to thank you for working through the prototype with me today. Your insights will be very helpful as we move forward.
- We’re almost done, but I want to talk with you briefly about your general impressions, what you liked, didn’t like… that kind of thing.

1. So, how was using the app for you?
2. Were there any specific features or functionalities of the app that you found particularly useful?
3. How would you rate the overall PAUSE app for ease of use?
4. How did you find the navigation – was it easy/hard to find your way around?
5. How understandable did you find the instructions and text within the app?
6. Was there anything you found especially confusing or difficult to use?
7. How about the amount of information and sick-day medication support the app provided – too much? Not enough?
8. Tell me about your level of trust in relying on the app’s recommendations?
9. How might you describe the PAUSE app to a friend or family member who might benefit from it?
10. Is there anything else you would like to tell us about the PAUSE app that we didn’t already talk about?

- Thank you for your time today, we’ve learned some great things about the PAUSE app and your experiences with it that will help us make it even better.
- We would like to thank you by giving you a $25 gift card. For this, we will need your email address.
- **Record email address in separate document.**
