# Supplementary figures and images for: Using Human-Centered Design and Development to Create a Digital Sick Day Medication Guidance Application for People With Diabetes, Cardiovascular Disease, or Chronic Kidney Disease: Mixed Methods Study
Source: JMIR Form Res. 2025 Nov 27;9:e77240. doi: 10.2196/77240 (PMC12778901; doi:10.2196/77240)

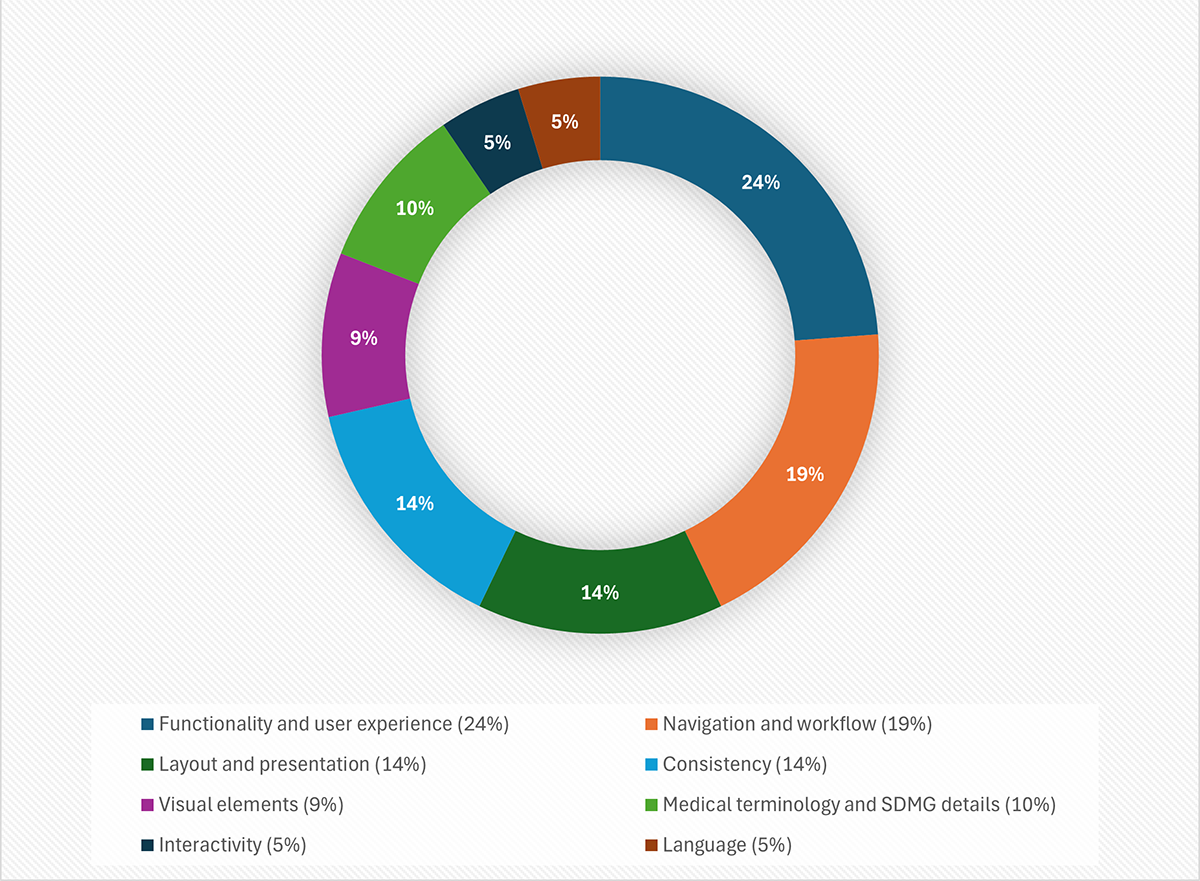

Supplement: Multimedia Appendix 2 [file formative_v9i1e77240_app2.png]

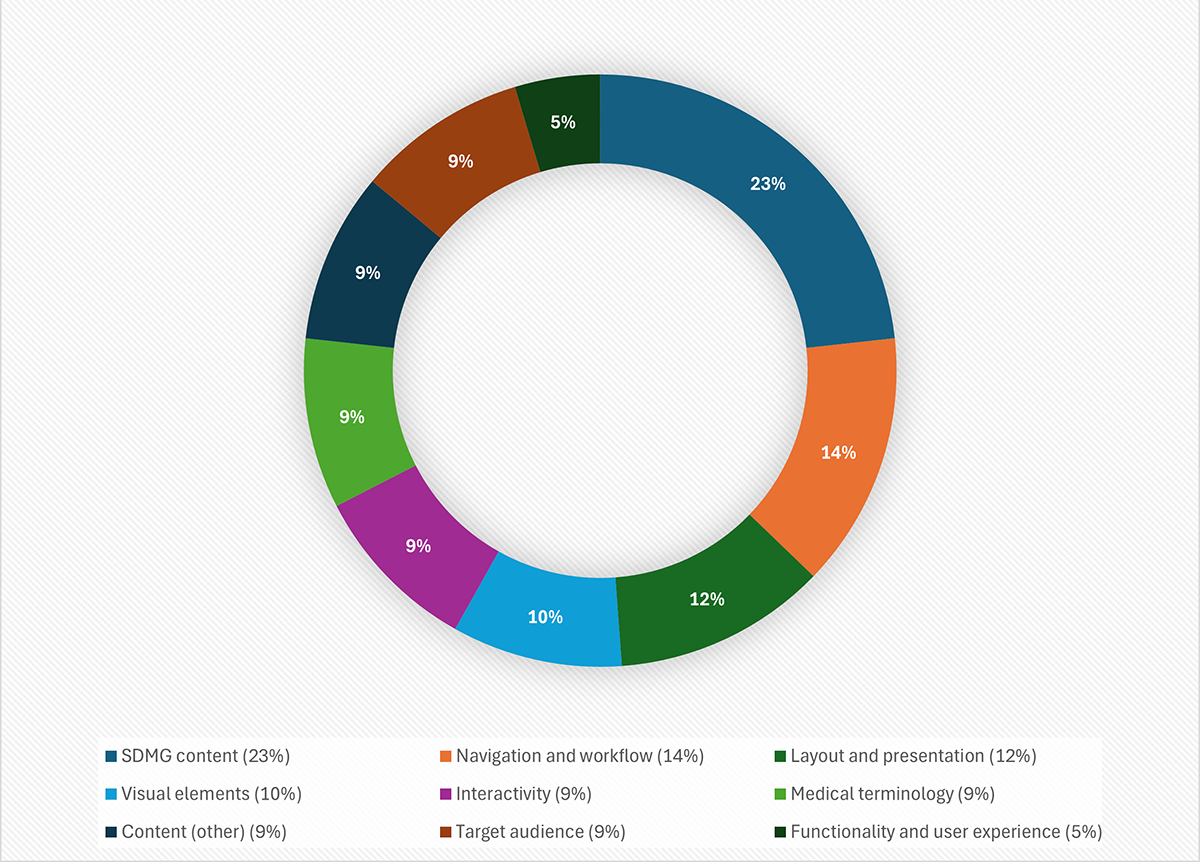

Supplement: Multimedia Appendix 4 [file formative_v9i1e77240_app4.png]
