# Supplementary material for: Using Human-Centered Design and Development to Create a Digital Sick Day Medication Guidance Application for People With Diabetes, Cardiovascular Disease, or Chronic Kidney Disease: Mixed Methods Study
Source: JMIR Form Res. 2025 Nov 27;9:e77240. doi: 10.2196/77240 (PMC12778901; doi:10.2196/77240)
Supplement: Multimedia Appendix 3 [file formative_v9i1e77240_app3.docx]

Multimedia Appendix 2: Heuristic evaluation results.

A total of 21 potential problems were identified, ranging from navigation and workflow issues to visual challenges and consistency concerns. Recommendations were provided where relevant.

## Navigation and workflow

### Workflow is incorrect in multiple areas (Priority Level 1)

**Issue Description:** Users experience navigation issues, as some selections do not bring the user to the correct page. Navigation issues within the app lead to incorrect medical advice, getting stuck in loops and being unable to progress.

**Further Details:**

- - - 1. On-boarding:
         1. When a user selects the option “I don’t know how my prescription gets filled”, they are brought to the beginning of the “Help us get to know you better” section and repeat this loop.
      2. Non-diabetes workflow:
         1. When the user selects "yes" for "Are you coping with managing your symptoms?", they are directed to the severe symptom page instead of receiving the correct medical advice, like pausing medications or continuing with their current medications.
         2. When answering the question, "Are you experiencing any new onset or worsening of symptoms?" the next screen is "Continue with your current medication advice," regardless of the user's selection. The medical advice provided does not reflect the user's response. Users should receive different medication recommendations depending on the symptoms they select.
      3. Diabetes non-insulin workflow:
         1. The screen does not advance on the question “Do you have any of the following home monitoring readings?”, regardless of selections or buttons pressed.
      4. Diabetes insulin workflow:
         1. The user is unable to access the “I am experiencing new symptoms” option when prompted to check in with the app.
      5. General:
         1. Pages have pre-selected options, which can mislead users.
         2. The back button is not functioning correctly. When users click the back button, they are directed to a random page that provides incorrect advice. Additionally, the back button sometimes loads pages with pre-selected choices, which can mislead users.

**Recommended Resolution:** To prevent catastrophic medical events, users must receive accurate medical advice. It is recommended to remove all preselected options, ensure that all pages advance along the correct pathway, verify that the back button functions as users expect, and ensure that users can access the right pages at the right time.

### Always end up on the onboarding screen (Priority Level 3)

**Issue Description:** After the onboarding process is completed whenever users open the app or press the home button, users return to the onboarding home page and have to sign in again.

**Recommended Resolution:** After completing onboarding, users should open the app directly to the home page without needing to sign in each time or view the rotating banner.

### No history or archive of the medical/medication advice, or symptoms (Priority Level 1)

**Issue Description:** There is no option for individuals to revisit the medical or medication advice provided once they leave the screen. This poses a problem if users need to refer back to the advice or share it with their healthcare provider. Additionally, any information entered is not saved, requiring users to re-enter their symptoms and details to receive proper instructions. This same issue arises when users are advised to call 911, 811, or visit a healthcare provider (HCP)/urgent care. Users are left without a way to access the information, and users may struggle to explain to an operator or healthcare provider why they are making the call or seeking urgent care, relying solely on their memory.

**Recommended Resolution:** Propose creating an archive section in the app where users can revisit their symptoms and the advice given. This would enable users to recall which medications they were advised to pause, when to call 811 or 911, or when to visit urgent care. Additionally, they could share this information with their healthcare providers if needed.

### The onboarding process is disjointed and confusing (Priority Level 3)

**Issue Description:** During the onboarding process, there is a lack of organization of the lists and options. The list alignment is off, and there is no consistency across the screens. Specifically, screen 1 of 5 lacks organization for the home monitoring options.

**Recommended Resolution:** Organize options into categories, for example, 'Screen 1 of 5', should have the options grouped according to body areas. This will help users identify relevant options easily as opposed to reading through the entire list.

## Layout and presentation

### Remove resources from the check-in page (Priority Level 2)

**Issue Description:** Users found the option to read resources when checking in with the app distracting. The app must be clear and concise, as users will access it when unwell. Unnecessary information and clutter should be removed from pages to ensure users can navigate the app easily during their illness.

**Recommended Resolution:** To improve clarity and the user experience, remove unnecessary information. It is advisable to delete resources from the check-in page and create a dedicated resources page that users can access at their convenience.

### The app has dead ends and lacks user feedback (Priority Level 2)

**Issue Description:** At the end of the onboarding process, there is no indication that it is complete, leaving users unsure if they have finished or if there are additional steps to take. Additionally, when users receive advice from the app, there is no option to exit the screen, no "X" button or "OK" button. These issues can lead users to believe they are stuck on a screen.


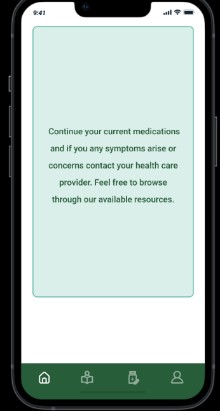


**Recommended Resolution:** The app should avoid dead ends; users must have the ability to exit pages or have options to return to the home page or the previous page. Additionally, it is important to provide user feedback so that users know when they have completed a task and what is expected of them. Some suggestions include showing a "Thank You" message on completion pages, providing a "Home" or "OK" button, or offering an option to close the page.

### Important text or information gets lost in blocks of text (Priority Level 3)

**Issue Description:** Important information can often be overlooked when presented in large blocks of text without proper highlighting. For instance, in the diabetes insulin pathway, the text states, “It is advised that you reduce your insulin doses until your blood glucose levels return to normal. If low blood sugar levels persist, please contact your healthcare provider.” The key point here is when to reach out to your healthcare provider.


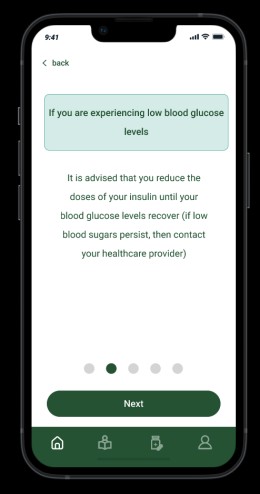

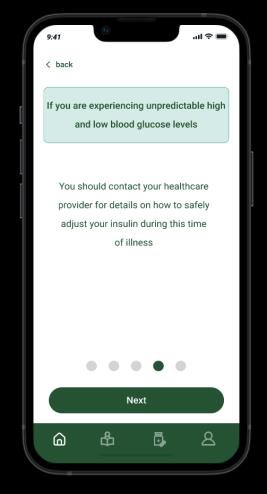

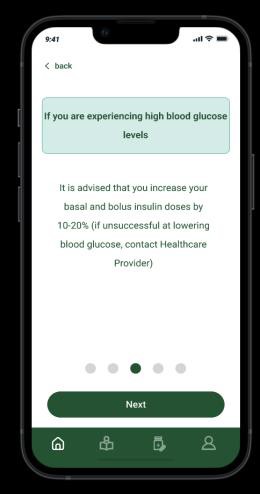


**Recommended Resolution:** Important information or calls to action should be clearly separated from large blocks of text by placing them on their own line, without using brackets. Ensure that text formatting and presentation of information is consistent across all screens.

## Visual Elements

### Confusion regarding icons displayed in the navigation bar (Priority Level 3)

**Issue Description:** Users are confused about the icons in the bottom navigation bar and are uncertain about their destination within the app.

**Recommended Resolution:** Enhance the clarity of the icons by using more familiar designs. Additionally, consider adding a ‘tooltip’ resource to explain the icons to users.

### Red, Green colour blindness issue with colour selection in the app (Priority Level 3)

**Issue Description:** The app uses red and green colours to highlight different decisions and important areas. People who are red-green colourblind may face challenges using the app effectively.

**Recommended Resolution:** Update the branding and colours to match the PC health app. Ensure visuals are colourblind-friendly by increasing the contrast between colours, utilizing a colourblind-friendly palette, and incorporating icons, bold fonts, and directional arrows to highlight important areas.

## Functionality and User Experience

### Rotating Banner is difficult to read and takes too long (Priority Level 3)

**Issue Description:** The text on the sign-in and sign-up page scrolls continuously, making it impossible for users to pause and read the information at their own pace. Additionally, the banner includes visual indicators at the bottom of the page that suggest users can interact with it—such as advancing, clicking through screens, or going back. The inability to skip a recurring banner or to read all the displayed information can cause frustration for users. Furthermore, inconsistent visual cues can lead to confusion when navigating the app.

**Recommended Resolution:** Allow users to read the information displayed at their own pace by removing the scrolling banner and adding "Next" buttons. Eliminate the circles at the bottom of the page, replacing them with the "Next" button. Additionally, do not show the banner every time a user logs into the app; instead, place that information in the resource section so users can access it whenever needed.

### Communication choices during on-boarding (Priority Level 3)

**Issue Description:** When selecting their notification preferences, users would like the option to choose both text and email notifications, rather than being limited to one option. Additionally, to improve the onboarding workflow, users prefer to complete their profile questions before choosing their preferred mode of communication.

**Recommended Resolution:** Allow users to choose both text and email, or add an option for "text and email." Move the notification options screen to the end of the onboarding process to avoid interruption.

### Promote user engagement (Priority Level 2)

**Issue Description:** The language used in the app does not encourage user engagement. For instance, when a user selects “I feel the same as normal,” the app responds with “Contact your health care provider if any symptoms arise.” Similar messaging appears on check-in days. If users are not motivated to interact with the app, they may stop using it altogether.

**Recommended Resolution:** Create messaging that encourages user engagement. For example, instead of saying, "Contact your healthcare provider if any symptoms arise," rephrase it to: "Check in the next time you're feeling worse than usual." Similarly, for check-in days, change "Day 1 Follow-up" to: "Tap here to check in today.

### Ensure the app is tailored to the user. (Priority Level 3)

**Issue Description:** During the onboarding process, users are asked about specific medications they take and whether they perform home measurements. When users enter their symptoms, they are asked these same questions again. This repetition can be frustrating and suggests that the app is not tailored to their individual needs.

Further Details:

- - - 1. In the insulin general advice pop-up, there is a statement that says, 'if you take insulin.' However, the user previously indicated that they take insulin, which is why they are guided through the diabetes insulin workflow.
      2. During onboarding, users are asked if they use specific home monitoring devices. This question is repeated after they enter their symptoms.
      3. When a user indicates that they measure their ketones during onboarding, they are asked whether they use urine, blood, or both methods. However, when it's time to enter their ketone measurements, they are always presented with options for both blood and urine. The ketone measurement options should correspond to the responses given during onboarding

**Recommended Resolution:** Users should only be directed to the insulin advice page if they indicate that they take insulin, so the phrase "if you take insulin" is unnecessary. After users have entered their symptoms, they should not be prompted to answer what home devices they use; instead, they should be asked questions about their device measurements. These adjustments will help reduce redundancy and user frustration. For questions regarding ketones, ensure that the inquiries reflect the type of ketones users measure, or provide an "n/a" option.

### The design of the “My medications” page is cluttered and confusing. (Priority Level 3)

**Issue Description:** When users navigate to the "My Medication" page to make edits, they find the layout challenging to read due to the overwhelming amount of information presented. It is not clear whether users can select individual medications for editing or if they are required to edit the entire page.

**Recommended Resolution:** Medications should be listed clearly, and the "verified" label should be removed from public view. The 'verified' statement is intended for pharmacists and the research study team; it serves as confirmation that the listed medication is appropriate for the user. Each medication listed should include its own edit icon, allowing users to know that they can modify individual medications without needing to edit the entire page.


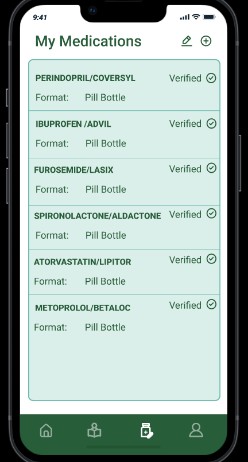


## Interactivity

### Users can turn all buttons on or off even if it is an either-or scenario (Priority Level 3)

**Issue Description:** When users respond to questions using "Yes" or "No" buttons, they can click on both responses, even if the question specifies that only one answer is allowed. It is noted that this could potentially be a Figma issue.

**Recommended Resolution:** When a response requires only one choice, ensure only one option can be selected. If this is a Figma issue, make sure the final design reflects this.

## Medical terminology and SDMG details

### Users are confused about medical advice for symptoms. (Priority Level 3)

**Issue Description:** Users often feel confused about why different symptoms come with varying medical advice. They may not understand the reasons for contacting a healthcare provider (HCP), what "sick day" medications are, or why they should pause certain medications. While this app provides a wealth of health information, it frequently fails to clarify why users should follow the given advice. This lack of explanation can lead to reduced credibility and trust in the app.

**Recommended Resolution:** Suggest creating a FAQ resource that explains why users need to contact their health care provider, what constitutes a sick day, and why these medications should be paused.

### Missing information to provide proper NSAID advice (Priority Level 3)

**Issue Description:** During the onboarding process (screen 4/5), users are asked if they take NSAIDS, this is posed as a yes or no question. If users select yes, they are not asked how often they take NSAIDs. This may lead to incorrect medical information being provided to the user.

**Recommended Resolution:** Make sure that the onboarding process records how frequently users take NSAIDs (none, rarely, occasionally, or regularly), so that the app can offer appropriate advice. If a user selects "rarely" or "occasionally," NSAIDs should not be included as a medication to pause on sick days. However, if a user selects "regularly," NSAIDs should be listed as a medication to pause.

.

## Language

### “Educational Resources” as a title can add pressure (Priority Level 3)

**Issue Description:** On the "How are you feeling today" pages, the phrase "Educational Resources" may come across as paternalistic and could create a sense of top-down pressure on the user. This wording suggests that the user needs to be educated or implies that they lack knowledge about their medications or illness.

**Recommended Resolution:** Change the heading to "Resources" to reduce top-down pressure, as this term indicates that users can access additional information as needed.

## Consistency

### Design of medication advice page (Priority Level 2)

**Issue Description:** On the "Medications to Stop" page, the display of listed medications resembles selection buttons. This design causes user confusion and is inconsistent with how items are listed on other pages.

**Recommended Resolution:** Ensure the medications are listed in a way that does not suggest they can be selected. Maintain consistency in how lists and selection options are displayed throughout the app.


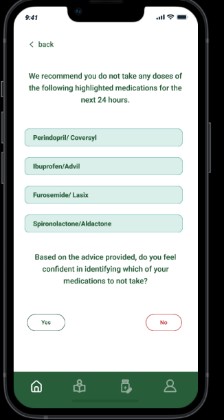


### Display of the user’s name varies across screens (Priority Level 3)

**Issue Description:** The user’s name is displayed on some screens and not on others.

**Recommended Resolution:** Only display the user's name on their profile page, as well as on the check-in and follow-up screens. User does not need their name on all pages.

### App design overall lacks consistency (Priority Level 1)

**Issue Description:** There is a lack of consistency throughout the app, including page designs, button appearances, navigation between pages, and the use of icons. This inconsistency can confuse users and hinder their ability to develop an efficient mental model of the app and its functionalities.

**Further Details:**

1. Some pages include a "Next" button, a "Submit" button, or automatically jump to the next page.
2. Within the app, various styles are used to indicate that there are multiple screens to navigate, such as coloured dots and numbering (e.g., 1/#). Also noted during the onboarding process, the screen numbers are incorrect, with "3/5" being repeated.
3. Text sizes and alignments vary throughout the app; sometimes the text is left-aligned, justified, or centred.
4. The Day 1 follow-up screen does not match the overall design of the app.
5. For some selection options, a plus sign is used. Generally, plus signs indicate that more information is available or that a section is expandable.
6. Users find it difficult to undo actions, and there is often no indication that an action has been completed. This can leave users feeling stuck or make the app seem unpredictable.
7.
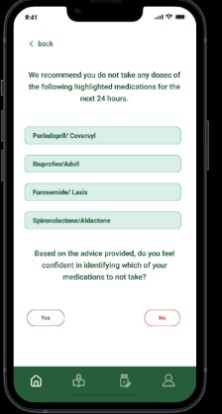

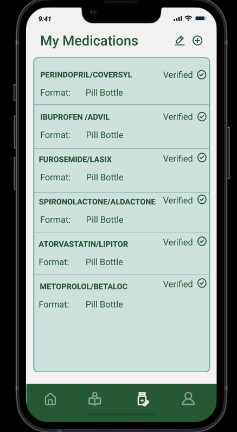
In the "My Medications" section, the naming convention for medications is inconsistent. Medications should consistently be listed as "Brand Name/Generic Name."
8. On the pages where users are asked for their systolic and diastolic numbers, only the first statement clarifies the systolic number is the top number. Each instruction should specify whether the user is looking at the top or bottom number.


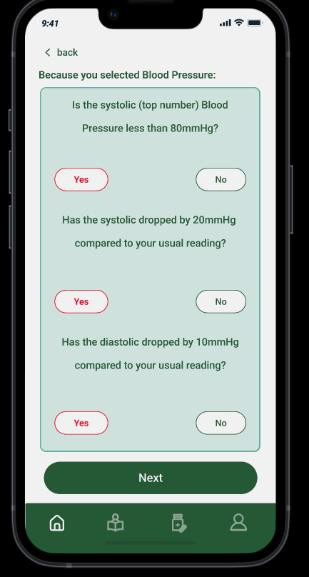


9. A similar design is used to indicate information, selections, and lists, which leads to confusion among users about whether they are reading a list, making a selection, or unsure of what action to take in the app.


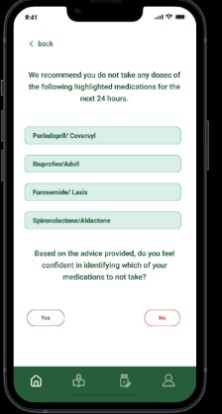

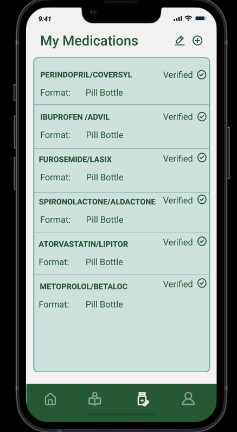

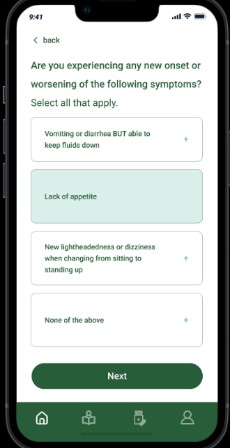


**Recommended Resolution:** To ensure a consistent overall look and feel of the app,

consider the following guidelines:

- Use the same design for navigation elements, such as the "Next" button, to move to the next screen.
- Apply a uniform design for displaying multiple pages within a section.
- Maintain consistent font style and alignment throughout the app.
- Ensure that all pages adhere to the PC style and branding guidelines.
- Utilize radial buttons for all selections; remove plus signs and check marks as selection indicators.
- Implement effective feedback mechanisms so users are informed about their actions and how to proceed to the next step.
- List all medication names written as brand name/generic name, ensuring that generic names are written in all lowercase.
- Clearly indicate whether blood pressure options refer to the top or bottom number.
- Correct any spelling and grammatical errors, and ensure that design elements (buttons, selections, etc.) are consistent throughout the app.
