# Supplementary material for: Using Human-Centered Design and Development to Create a Digital Sick Day Medication Guidance Application for People With Diabetes, Cardiovascular Disease, or Chronic Kidney Disease: Mixed Methods Study
Source: JMIR Form Res. 2025 Nov 27;9:e77240. doi: 10.2196/77240 (PMC12778901; doi:10.2196/77240)
Supplement: Multimedia Appendix 5 [file formative_v9i1e77240_app5.docx]

# Usability testing

A total of 43 usability issues were identified, and recommended resolutions were provided where possible. Overall, the testing results indicate a strong positive reception and an expected usefulness of the PAUSE app. However, the app's purpose and target audience should be refined to maximize its effectiveness.

## Navigation and Workflow

### Page 4 of 5 (onboarding) doesn’t auto-scroll to the top of the page

**Issue Description:** When users reach page 4 of 5 in the onboarding section, the page loads with the middle section displayed. This can be confusing because the information does not align with what users saw on the previous screen, and they may not realize they need to scroll up to view the content correctly.

**Recommended Resolution:** Ensure that all pages load correctly

### Missing “Next” button leads to unexpected and surprising app behaviour

**Issue:** A "Next" button was missing on one of the pages, leading to unexpected and surprising behaviour within the app. This absence can create a sense of lost control for the user, adding to their stress in what is likely already a challenging situation.


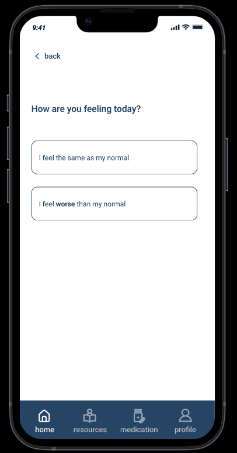

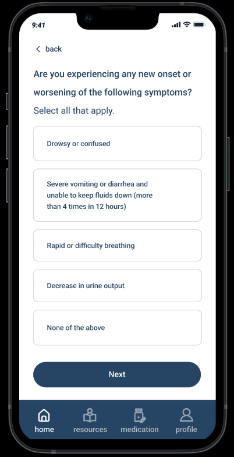


**Recommended Resolution:**

Ensure that all pages with steps in a workflow include the “Next” button.

### App does not prompt users to check junk mail for the verification email

**Issue:** Users noted from previous experiences that notification emails sometimes end up in their junk folder, but they may not always think to check there.

**Recommended Resolution:**

After onboarding, a notification should appear prompting users to check their junk folder if they have not received the email.

###### Fear that a clinician would not discuss insulin adjustments over the phone

**Issue:** Users are advised to reach out to their health care provider if they are experiencing low or high blood glucose levels and have not been successful in making adjustments. Many users lack confidence that their health care provider will offer any advice over the phone and are unsure about the type of guidance they would receive.


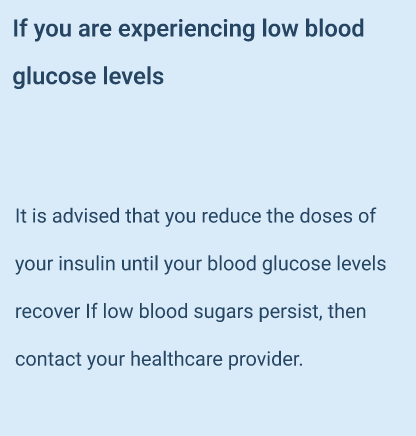

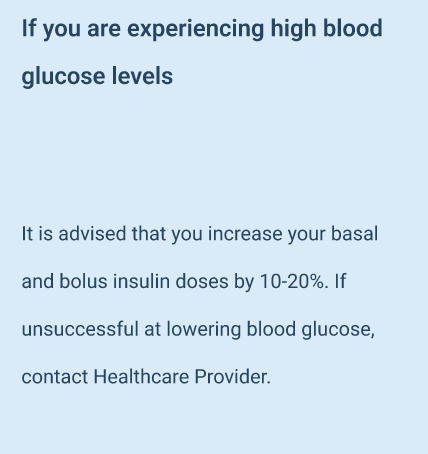


**Recommended Resolution:** Ensure that if users are advised to call their HCPs in any situation, the app advises them that this type of guidance can be given over the phone. Consider adding statements like, “Depending on your situation, your care provider may be able to help you adjust your insulin dosages over the phone” for reassurance. It is also recommended that HCPs remind patients that dosage changes can be communicated over the phone.

###### Users are taking notes on proactive steps to prepare for sick days, such as keeping Gatorade at home for future illnesses (learning opportunities)

**Issue:** Users find the self-management advice helpful, but they can only access this advice during a sick day event. They would like to proactively prepare for a sick day.


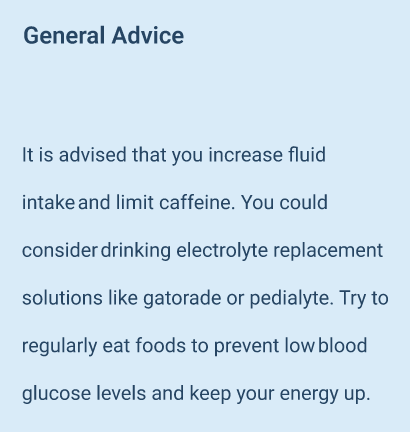


**Recommendations:**

Enhance the resource page by emphasizing self-management tips for mild to moderate symptoms. Make archived advice more noticeable, allowing users to reference past symptoms and advice easily.

###### Users want their HCP aware sick days and advice provided by app for monitoring and follow-up

**Issue:** Users are concerned that their health care provider(s) would be unaware of a sick day occurrence and the advice given to stop and start medications. Users want their HCP to be alerted and prompted to reach out to them for follow-up if necessary.

**Recommended Resolution:** The Research team will review this issue, as general practitioners expressed reluctance to interact with notifications from the app and follow up with their patients.

## Layout and presentation

### Text alignment and item grouping of items on page 1/2 “topics” and 1/5 “conditions”


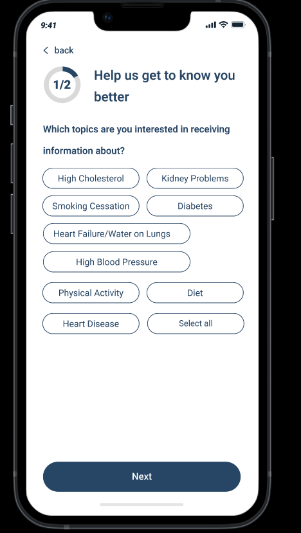
**Issue:** The alignment and configuration of the condition and topic during the onboarding process impede users from easily scanning the options.

**Recommended Resolution:** Suggest organizing the conditions either alphabetically or by their relatedness. Additionally, ensure the list is left-justified and convert the items into checkboxes instead of buttons.

### (Form Factor) The layout and design present challenges, especially for small (e.g., iPhone SE 2022) form-factor devices

**Issue:** Users with smaller phone screens faced difficulties in identifying the next button, as there were no visual cues such as a scroll bar to indicate there was more information. The cutoff point of the text also did not suggest that additional content was present beneath it.

**Recommended Resolution:** Add a scroll bar to the side of the screen to indicate scrolling is required. Ensure that key elements are at least partially visible on the “fold” of a smartphone screen to hint to the user that there is more below.

### Ensure responsiveness to changing aspect ratio (portrait versus landscape)

**Issue:** Some users prefer to interact with their phones in landscape orientation which could create layout issues.

**Recommended Resolution:** Ensure the app is responsive to changing device orientation and that the screen layout continues to be usable and aesthetically sound regardless of orientation.

### (Form Factor) Narrow portrait screen makes options difficult to read


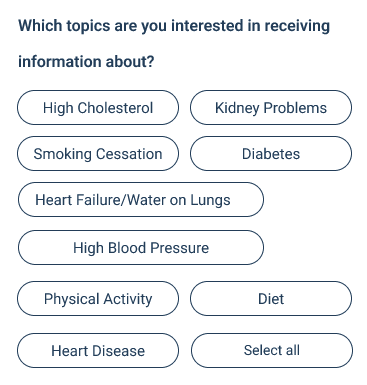
**Issue:** Some content is difficult to read in a narrow portrait layout, especially when lists aren’t organized in descending order.

**Recommended Resolution:**

Ensure text is succinct and left-justify lists with one item per row to support scanning.

### Medication dosage and pictures should be included in the list of medications

**Issue:** Users expressed challenges in identifying medications solely by their names and raised concerns regarding the absence of dosage information. They consider both of these details essential when sharing their medication lists.

**Recommended Resolutions:** Recommend adding pictures of user medications, similar to the example below. Ensure that the medication list contains details regarding the dosage and frequency of each medication.


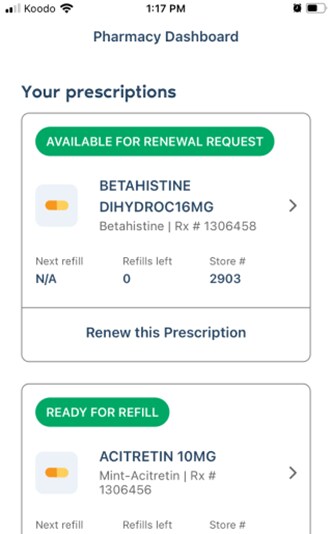


## Visual Elements

### (Icons) Ensure clarity of small icons

**Issue:** Some icons used within the prototype were not recognizable by several users, leading to misunderstandings of what different features within the app are.


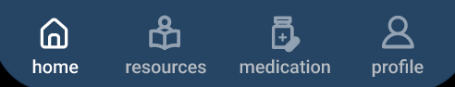

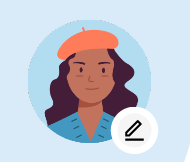

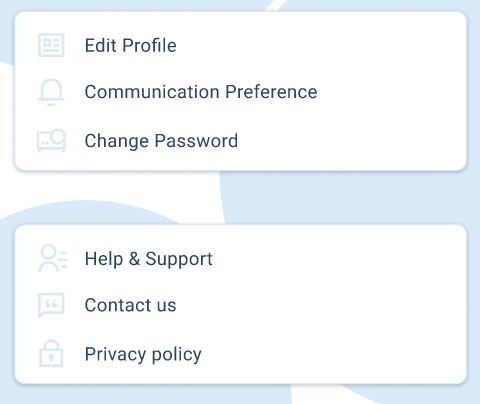


**Recommended Resolution:**

Use icons sparingly, and icons used should be clear and adhere to WCAG 2.0 accessibility guidelines. To promote a better understanding of functionality, use text instead of icons. For example, instead of using a pen or plus icon, the terms “Edit” and “Add” should be displayed.

### (Colours) Ensure sufficient contrast for text colours

**Issue:** Some text was very light in colour and difficult to read.


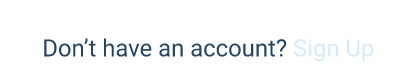
**Recommended Resolution:** Ensure sufficient contrast between background and text using the WCAG 2.0 accessibility guidelines.

### (Colours) Use colours and shapes consistently


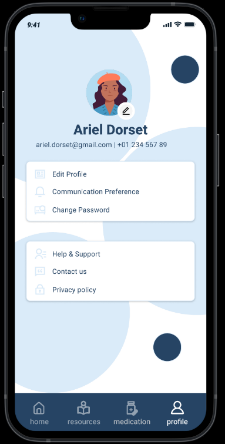
**Issue:** In certain instances, a dark blue circle serves as an indicator for a clickable element, while on other pages, it is utilized purely for decorative purposes. This inconsistency has resulted in user confusion, as individuals anticipated that the dark blue circles would have a functional role.

**Recommended Resolution:** Maintain consistent colour usage throughout the app to prevent any possible confusion. Refrain from applying buttons or link colours to elements that are not interactive.

### (Colours) Use red sparingly and intentionally

**Issue:** Users assume red button colours indicate an alert, danger, or to stop. Users also find button colours are inconsistent.


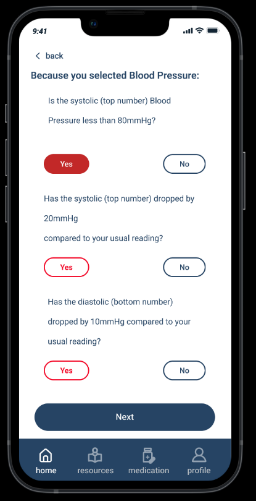

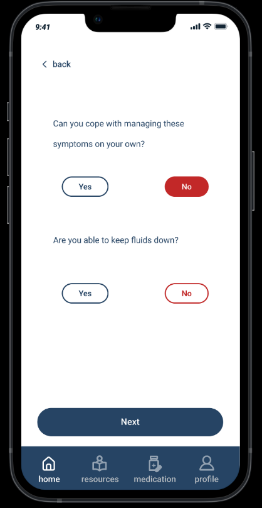


**Recommended Resolution:**

Use red sparingly and intentionally, and reserve it for error messages. Ensure button colours are applied consistently and match the context.

## Functionality and user experience

### Face and fingerprint recognition may not work well for users.

**Issue:** Users shared their personal experience with apps and facial recognition not working if they wear glasses. Some users also mentioned that they were unsure how to use fingerprint recognition.

**Recommended Resolutions:** Ensure multiple ways to log on to the app, and consider removing face and fingerprint recognition altogether.

### Concerns around the workload of manual medication entry

**Issue:** Users have raised apprehensions about the process of inputting their medications. They believe that this task could lead to potential errors and would require a significant amount of time. Additionally, users indicated that managing their chronic conditions already involves numerous daily responsibilities, and this feature would further increase their burden.

**Recommended Resolution:** Have the app retrieve the list of medications from relevant pharmacies, NetCare or an alternative platform.

## Interactivity

### App lacks a “Select all” options

**Issue:** When a list contains numerous options, users prefer the ability to "select all" items at once. Users found selecting items individually to be a tedious task.

**Recommended Resolution:** Add a “Select all” option to lists where appropriate.

### Comfort with a computer versus smartphone

**Issue:** Certain users prefer using their smartphones primarily for calls and occasional texts, and may not be as comfortable with touchscreen interactions. These users have expressed a preference for working on a computer with a mouse, as it offers a more familiar and comfortable experience.

**Recommended Resolution:** If possible, provide a desktop/web version of the app for users who prefer this option. Additionally, ensure that the app functions properly in a horizontal orientation for those who prefer using their devices this way.

### Lack of checkboxes for selection options leads to user confusion

**Issue:** Users find it unclear whether they have selected options when only a colour change indicates selection. They would prefer the use of checkboxes to clearly select and deselect options as needed.


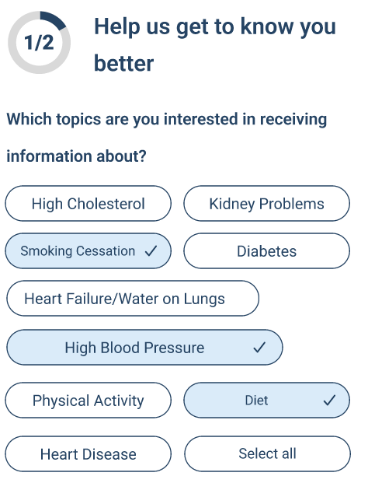
**Details:**

**Recommended Resolution:** Make options into a checklist

### “X” Close button does not always function

**Issue:** The “X” close button did not always work creating user frustration

**Recommended Resolution:**

Ensure interactive elements (such as the “x” close button) work as expected.

## Medical terminology

### Typos in Diabetes Canada handout

**Issue:** Users have identified typos and formatting issues in the Diabetes Canada handout. Although this handout is not a PAUSE product, such errors can erode users' trust in the app. For example, "1/3 cup juice" is not formatted correctly, and "milk" has an asterisk without any explanation of what the asterisk signifies.


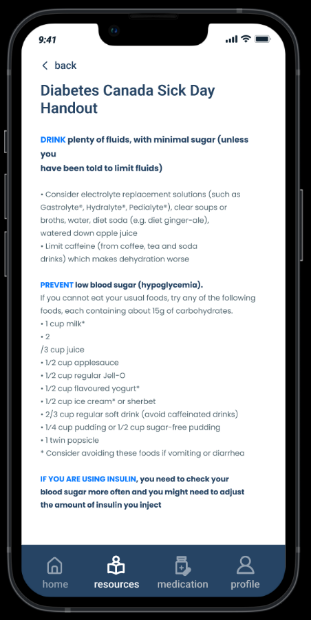


**Recommended Resolution:** Ensure that all content, resources, and handouts are meticulously checked for typos, grammatical errors, and formatting issues, regardless of their source.

### (Terminology) Which blood pressure number is systolic?


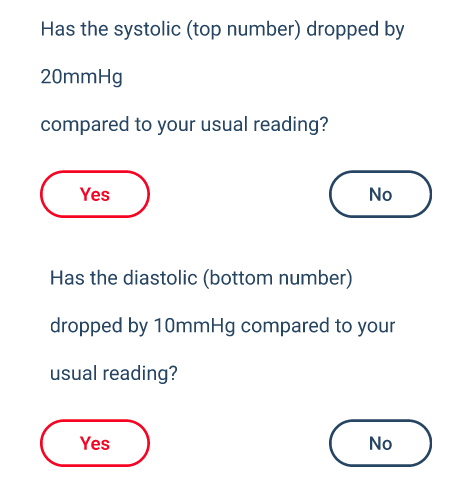
**Issue:** Some users have difficulty remembering which blood pressure number is systolic (top) and which is diastolic (bottom). Additionally, the units of measurement (mmHg) can be confusing, as users may only focus on the numbers within the ideal range (e.g., 120/80 vs. 120mmHg/80mmHg).

**Recommended Resolution:** To support cognitive recognition, include "top number" or "bottom number" in brackets whenever diastolic or systolic is mentioned. Ensure this is consistent throughout the app. For example, you could use "Has the top number (systolic pressure)..." or "Has the bottom number (diastolic pressure)...".

### (Terminology) Not sure what NSAID means


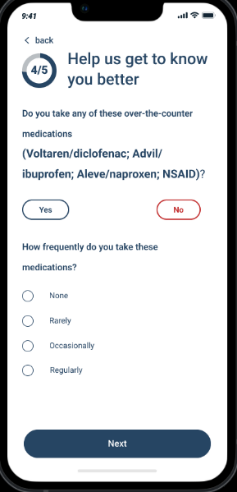
**Issue:** When users encounter the term NSAIDs followed by generic and brand names in brackets, they are often unsure whether to select NSAIDs because they do not understand what the term means.

**Recommended Resolution:**

Remove the term "NSAID" from the options, or clarify it by using the statement: "Do you take any of these over-the-counter medications (non-steroidal anti-inflammatory drugs)?" This will help users understand the term better.

### (Terminology) Unsure what ketones are

**Issue:** When presented with a scenario, some users may be unfamiliar with ketones, as they do not typically track this information. This situation can arise during onboarding if users accidentally select an option they do not monitor at home.

**Recommended Resolution:**

Consider implementing a confirmation page during onboarding to verify that the selection choices accurately reflect what users monitor at home. This will help ensure users do not accidentally select options they do not track.

## SDMG content

### (Language) What does “cope with symptoms” mean?

**Issue:** Some users find the question "Can you cope with managing these symptoms on your own?" difficult to interpret. The term "coping" is complex and vague, which might cause more stress than reassurance for app users.


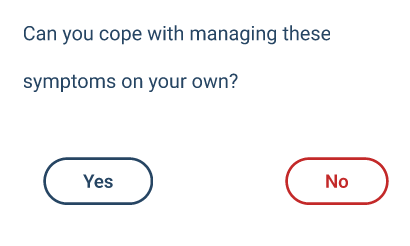


**Recommended Resolutions:**

Consider rephrasing the question to be more specific and less ambiguous. For example, instead of asking, "Can you cope with managing these symptoms on your own?", you could ask, "Are you able to manage these symptoms by yourself?" This wording is clearer and may reduce stress for users.

The context of "feeling better" and "feeling worse" in relation to living with chronic diseases is unclear


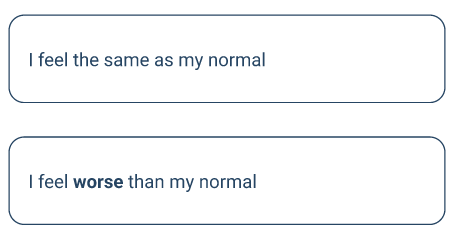
**Issue:** Some users expressed confusion or apprehension regarding the statements "feeling better" or "feeling worse." These phrases are subjective and context-sensitive, particularly for individuals living with chronic diseases, who may never feel "better" compared to their pre-disease "normal."

**Recommended Resolution:**

Ensure language clarity and avoid vague and ambiguous statements that can be misinterpreted.

### Lack of user recall for previous symptoms entered

**Issue:** During the app flow, users are asked to compare their symptoms with those from the previous day. However, some users may struggle to recall their symptoms from the day before. This is especially true for users who are not feeling well, as memory issues and confusion can be common. It's important to recognize and support these challenges.


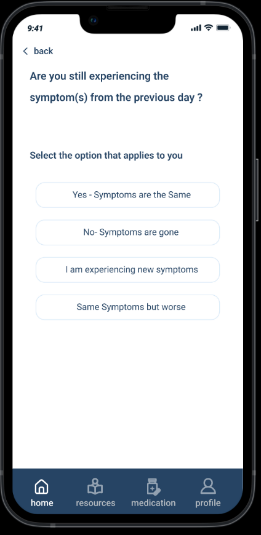


**Recommended Resolution:** Users should have access to their previous symptoms.

## Users are startled by instructions to go to the hospital and are unsure why they are being told to call 911?

**Issue:** Users are unclear about why they are being prompted to call 911 or go to urgent care. Users felt that they wouldn’t be able to comfortably explain to the 811/911 or the urgent care doctor why they were calling or there. The advice was found to be concerning and startling by users.


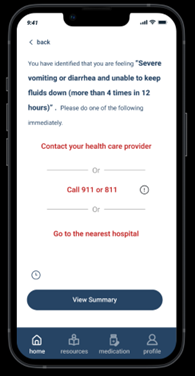


**Recommended Resolution:**  At the beginning of the app, clearly inform users that they might receive advice to go to the hospital or seek immediate medical attention based on their symptoms. This will help prepare users for such recommendations.

Provide clear and detailed explanations within the app about why users are being prompted to call 911 or go to urgent care. This should include specific symptoms or scenarios that warrant such advice.

Add a button labelled “Information for Health Care Providers” to the page. This page will contain specific information that patients can provide to the ER doctor, helping them explain why they are at urgent care.

### Type of pill bottle not specified

**Issue:** Users noted that specifying their preferred type of pill bottle would be useful information, as some individuals have difficulty opening standard pill bottles.


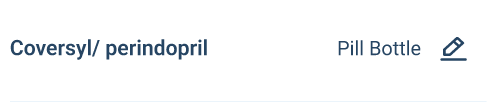
**Details:**

**Recommended Resolution:** Include additional information on the type of pill bottle a medication is dispensed in to ensure all relevant details are accurately captured. This can help accommodate users, especially those, who may have difficulty opening standard pill bottles.

### Limited resource topic areas

**Issue:** Users observed that most resources were tailored for people with diabetes, and noted the absence of resources for individuals with kidney disease. This lack of inclusivity may lead users to question whether the app is intended for them.

**Recommended Resolutions:** Include resources and information for all the conditions the app addresses. Ensure that the app’s splash screen clearly communicates the intended audience.

### The range of medical conditions seemed limited


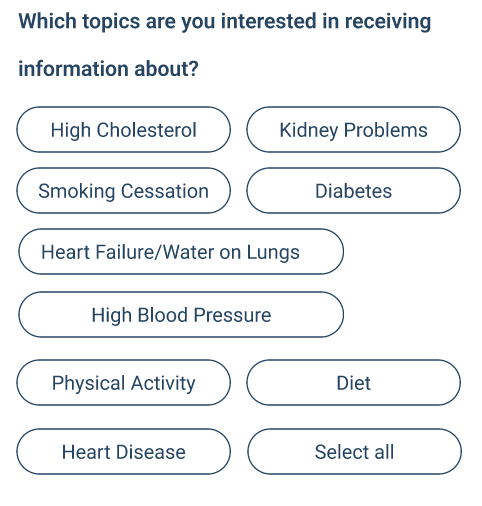
**Issue:** Users felt that the app's guidance was based on conditions rather than medications. They were concerned that their symptoms might be related to a condition or topic not covered by the app.

**Recommended Resolutions:** Consider including additional chronic conditions/topics that may require SDMG. Clarify in the onboarding section and throughout the app that the focus is on medications to stop, not necessarily specific conditions.

### Medication frequency question is hard to answer

**Issue:** During the onboarding phase, users found it challenging to answer the refill frequency question because not all medications are refilled at the same time.

**Recommended Resolutions:** Remove this question, this information is not needed for the app to function.

### Are all resources in the app for sick day medication guidance?

**Issue:** Some users found the information in the resource section confusing, as they were unsure whether it was specific to SDMG or generic health/condition information.

**Recommended Resolution:** Consider clearly labelling the resources to indicate whether they pertain specifically to SDMG or are general health information. This will help users better understand and utilize the resources provided. Also, consider removing all non-SDMG resources to make the app more tailored.

### Context needed for what each symptom entails (i.e., lightheadedness is sudden and persistent, indicative of low BP)

**Issue:** Users have reported that some symptom-related questions reflect their usual condition experiences, leading to confusion. For instance, light headedness is common among those with blood pressure issues, so regular occurrences of this symptom may not be concerning. Additionally, individuals with hypertension often experience significant variations in blood pressure measurements, making it challenging to answer questions like, "Has the systolic (top number) dropped by 20 mmHg compared to your usual reading?" as such fluctuations are normal. These circumstances can confuse and erode trust in the app.


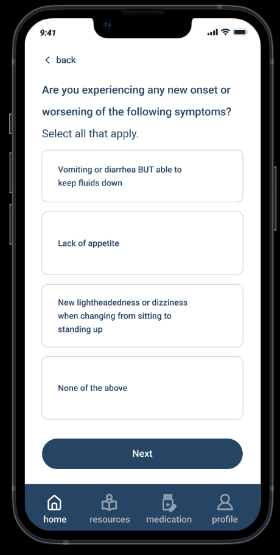


**Recommended Resolutions:** Clarify these questions are intended to identify symptoms that are sudden in onset or significantly different from the user's normal experience.

### Blood pressure measurement questions assume users have a home device

**Issue:** Users have noted that the app's questions about blood pressure seem to assume that they can measure their blood pressure at home. This may not always be feasible, especially when users are feeling unwell.

**Details:**


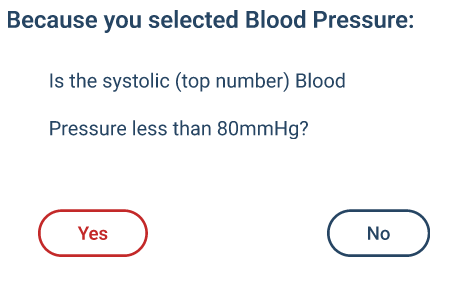


**Recommended Resolutions:** Adjust the question to include, "if you have a blood pressure device at home." Additionally, providing an "N/A" option for users who cannot measure their blood pressure would ensure more accurate and inclusive responses.

### (Learning opportunity) Users don’t understand why they need to stop their medications

**Issue**: Users have expressed a desire to understand why certain medications should be stopped when they are sick. There is a concern about discontinuing medications that their doctors have advised them to take consistently.

**Recommended Resolution:** Suggest providing a clear explanation about the reasons for stopping specific medications during an illness to help alleviate fears and ensure users feel more confident in following medical advice. This information should be based on drug classes, and each drug class should have it’s own statement. To ensure users start medications again, add in a note that Pausing medications is temporary and medications should be started again in 3 days.

## Content

### (Terminology) Confusion around “Communication preference”

**Issue:** Some users have misunderstood the term "communication preference," thinking it referred to language preference (e.g., French or English) rather than the method of communication (e.g., text or email). This indicates that the terminology used for communication preferences may be too vague and could benefit from clarification.


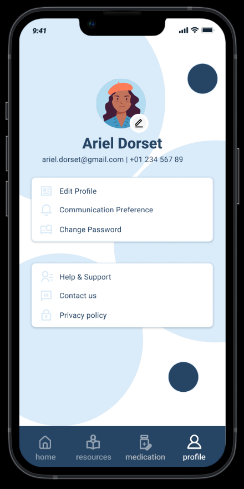


**Recommended Resolution:** To avoid confusion, it would be beneficial to replace the term "communication preference" with "notifications" in the user profile. List the options as "email," "text," and "email & text." Additionally, during onboarding, remove the term "communication preference" and start the screen with, "Please let us know how you prefer to be notified." This approach should help clarify the options and prevent misunderstandings.


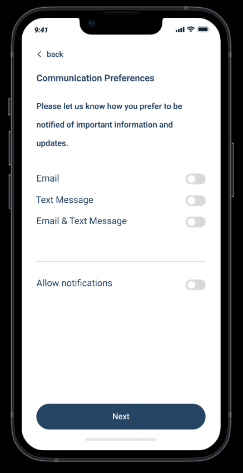


### Users need reminders of their previous symptoms

**Issue:** Users appreciate the app’s check-in page, which asks if they can cope with their symptoms, reminders to resume medications, and overall asks how they are feeling. However, users have noted that recalling previous symptoms can be challenging, which makes it difficult to answer questions or interact with the app effectively.

**Recommended Resolution:** Including a brief summary of the user's last check-in or a history of recent symptoms could be very helpful. This feature would aid their memory and enhance their overall experience with the app by making it easier to recall and report their symptoms accurately.

### Users forget the purpose of the app

**Issue:** The app currently lacks introductory information to explain its purpose and what users can expect. Placeholder text (Lorem ipsum) is displayed instead, causing confusion among participants about the app's purpose and whether they need sick day medication guidance. This confusion may deter individuals from using the app if they are unsure of its function or necessity. Additionally, users mentioned that they would only use the app when unwell, which could lead to forgetting its purpose due to infrequent use. Specific examples include:

- Participants with kidney issues found the app irrelevant because they were unaware that medications for their kidneys could be harmful during dehydration or fluid loss when sick.
- Use practical, real-life language to describe the app instead of just "PAUSE," as it doesn't clearly convey the app's purpose. A term related to disease management would be preferred.
- Participants who regularly use medication were unaware of the app's purpose and distrusted its advice, as they have been taking the same medication for years.
- There is no warning about the type of advice that will be given (e.g., being advised to go to the hospital based on input symptoms).

**Recommended resolution:** To enhance user understanding and engagement, the app should include the following:

1. **Introductory Text**: Provide clear and concise information about the app's purpose, target audience, and expected benefits. This will help users quickly grasp the app's relevance to their needs.
2. **Guided Tour Screens**: Offer optional guided tour screens that demonstrate how to use the app, highlight key features, and explain how to act on the provided information. This will make the app more user-friendly and accessible.
3. **Support Materials**: Include a brief video and a handout that cover the same information as the guided tour. This caters to different learning preferences, ensuring all users can effectively understand and use the app.
4. **Clarification on Conditions and Medications**: Provide detailed information on conditions and medications that fall under Sick Day Medication Guidance (SDMG). This is particularly important for individuals with kidney disease, who may not realize that their medications are relevant to SDMG.

By implementing these elements, the app can better educate and support its users, making it more likely they will use it effectively when needed.

### (Terminology) Issue with language and terminology used

**Issue**: Some users were unaware of what caffeine is and did not realize that coffee and other drinks contain caffeine.

**Recommended Resolution:**  Avoid using the term "caffeine" alone. Instead, add examples in brackets to ensure users understand what is being referred to. For instance, specify that coffee (or tea, coke, or instant flavoured coffee) contains caffeine. Additionally, provide clear definitions and examples throughout the app to enhance clarity and understanding.

## Target audience

### Users with vision issues might have difficulty using the app

**Issue:** Several users found the text in the app too small, which could be problematic for users with vision issues, a common concern for the elderly and people living with diabetes.

**Recommended Resolution:** Increase the text size and provide options for text resizing within the app. Additionally, consider implementing a high-contrast mode to further enhance readability for all users. Ensure W3C Content Accessibility Guidelines (WCAG 2.0) are adhered to for contrast and text size.

### Users have concerns regarding data security and privacy

**Issue:** Some users misunderstand the implications of downloading and using the app, believing that avoiding online platforms means their data isn't stored digitally. Additionally, other users have concerns about their privacy and how the research team will handle their data.

**Recommended Resolution:** Provide clear information about how data is stored and managed, emphasizing that data is stored digitally even if users avoid engaging with online platforms. Include a section in the app that transparently outlines the privacy policy and data usage terms.

### Some users use more than one pharmacy

**Issue:** Some users use different pharmacies for various medications and are concerned about whether medications obtained from non-PC pharmacies fall under the Sick Day Medication Guidance (SDMG) and how that would be managed.

**Recommended Resolution:** Ensure that app resources have sections that clearly explain which medications fall under SDMG, regardless of the pharmacy they are obtained from. This will help users understand that the guidance applies to all relevant medications, not just those from PC pharmacies. Let users know that as of now the app is only available for PC app users.

### Lack of real-life practical language throughout the app

**Issue**: Users were unclear about the app's purpose, the meaning of "sick day medication guidance," and the phrase "feel worse than my normal" (e.g., confusion about symptoms like vomiting, especially if hungover).

**Recommended Resolution:** Revise the app's language to be more accessible to non-medical users. Avoid medical jargon and use plain language to ensure clarity. Include definitions for any necessary medical terms. Incorporate everyday examples to explain medical concepts. For instance, instead of saying "caffeine," specify "coffee, tea, cola, or energy drinks."

## Branding

### The look of the Pause logo was found to be confusing

**Issue:** Users did not recognize the logo as a pill; instead, they thought it resembled a house, mushroom, or other objects.

**Recommended Resolution:**
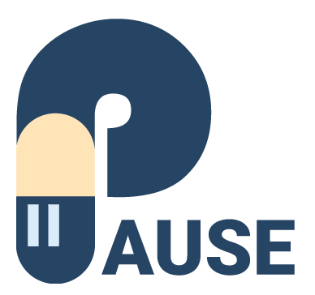
This was not an issue for all users, so the logo could be left as is or consider redesigning the logo to more clearly represent a pill.
